# From Self-Regulation to Function: A Systematic Review and Meta-Analysis of Randomised Controlled Trials in Autistic Children and Young People

**DOI:** 10.3390/children13101415

**Published:** 2026-10-15

**Authors:** Jacinta So, Yu-Wei Ryan Chen, Sarah Wilkes-Gillan, Lauren Parsons, Katherine Spender, Reinie Cordier

**Affiliations:** 1 Sydney School of Health Sciences, Faculty of Medicine and Health, The University of Sydney, Sydney, NSW 2006, Australia; ryan.chen@sydney.edu.au (Y.-W.R.C.); sarah.wilkes-gillan@sydney.edu.au (S.W.-G.); 2 Curtin School of Allied Health, Faculty of Health Sciences, Curtin University, Perth, WA 6102, Australia; lauren.parsons@curtin.edu.au (L.P.); reinie.cordier@curtin.edu.au (R.C.); 3 School of Communities and Education, Faculty of Health and Wellbeing, Northumbria University, Newcastle NE1 8ST, UK; 4 Department of Health & Rehabilitation Sciences, Faculty of Health Sciences, University of Cape Town, Cape Town 7700, South Africa

**Keywords:** autism, self-regulation, functional outcomes, randomised controlled trials, systematic review, meta-analysis, generalisation, intervention

## Abstract

**Highlights:**

**What are the main findings?**

- Self-regulation-focused interventions produced positive average effects on both self-regulation and separate child functional outcomes;
- Functional effects were substantially larger for outcomes directly aligned with intervention targets than for distal outcomes requiring generalisation, although distal effects remained positive.

**What are the implications of the main findings?**

- Intervention trials should distinguish proximal self-regulation outcomes from target-aligned and distal functional outcomes;
- Claims of intervention effectiveness should consider both comparator design and how closely measured functional outcomes align with what was directly trained.

**Abstract:**

**Background and Objectives**: Self-regulation is important for children’s learning, social participation and everyday functioning, yet intervention evidence for autistic children and young people is fragmented across intervention approaches and regulatory constructs. This systematic review and meta-analysis aimed to characterise and critically evaluate non-pharmacological randomised controlled trials targeting self-regulation in autistic children and young people and to quantify effects on both self-regulation and separate child functional outcomes. **Methods**: MEDLINE, PubMed, PsycINFO, CINAHL and ERIC were searched to 24 May 2026. Eligible studies were randomised controlled trials involving autistic children and young people aged 0–18 years that targeted at least one domain of self-regulation and assessed both self-regulation and child function. Risk of bias was assessed using RoB-2. Random-effects meta-analyses were conducted for self-regulation and functional outcomes, with moderator, meta-regression and sensitivity analyses examining sources of variation. Functional proximity and intervention-mechanism moderator analyses were exploratory and were not prespecified in the registered protocol. **Results**: Twenty-five studies involving 1655 randomised participants were included. Self-regulation improved significantly across 27 intervention–comparator effects from 25 studies (Hedges’ *g* = 0.605, 95% CI 0.454–0.756; 95% prediction interval 0.027–1.183). Functional outcomes also improved across 25 effects from 23 studies (*g* = 0.527, 95% CI 0.356–0.698; 95% prediction interval −0.158–1.212). Functional effects were substantially larger for direct/target-aligned outcomes (*g* = 0.761) than for distal/generalisation outcomes (*g* = 0.250; *Q*_β_ = 11.034, *p* < .001). The direct–distal contrast attenuated when restricted to active-comparator studies (*Q*_β_ = 3.770, *p* = .052), although distal effects remained positive. Comparator type moderated both outcome streams, whereas self-regulation construct coverage, age, intervention duration, target-skill application and explicit generalisation support did not significantly moderate effects. **Conclusions**: Self-regulation-focused interventions produce beneficial average effects on both regulatory and functional outcomes. Functional improvement is strongest when outcomes align closely with intervention targets, although smaller positive effects also extend to distal outcomes. Future trials should distinguish proximal regulatory change from target-aligned and distal functional outcomes and explicitly test pathways of generalisation.

## 1. Introduction

*Self-regulation* is a vital component of human development and everyday functioning [1] that involves the capacity to calibrate and adjust emotions, behaviours and thoughts to execute goal-directed actions [2,3,4,5,6,7]. Self-regulation develops most rapidly during early childhood [8,9,10,11] and continues into adolescence and early adulthood [12,13,14,15]. To date, numerous studies have established the association between greater child self-regulation abilities and improved academic performance, social competence, positive wellbeing and health-related outcomes [1,16,17,18]. Conversely, poor self-regulation abilities are related to a greater risk of mental and physical challenges [19]. These associations are particularly relevant for autistic children and young people, for whom differences across the emotional, behavioural and cognitive regulation domains may affect participation, learning and social functioning [20,21]. Consequently, a wide range of interventions has been developed to support self-regulation in autistic children and young people.

Self-regulation has been conceptualised in different ways across disciplinary studies, with related terms including self-control, effortful control, and self-management, as well as terms referring to specific self-regulation domains, such as behavioural regulation and emotion regulation [22,23,24,25]. Recent systematic reviews of self-regulation measures [22,23] reported considerable variation in the comprehensiveness of existing outcome measures due to diverse terminology and incongruous definitions. In addition to this, these discrepancies complicate the selection of appropriate interventions to address child self-regulation and the synthesis and review of existing interventions.

The contemporary literature proposes that the multi-dimensional construct of self-regulation comprises the following three domains: emotional, behavioural and cognitive regulation [22,23,24,25,26,27,28,29]. The *emotional regulation* (ER) domain is associated with managing and modulating emotions, including their experience, expression, and outward exhibition [12,30,31]. The *behavioural regulation* (BR) domain involves the ability to control behavioural responses, encompassing the capacity to initiate and inhibit actions and manage impulse reactions [32]. Finally, the *cognitive regulation* (CR) domain comprises mental functions involving attentional control [3], memory [25] and flexibility [33], along with management, awareness and adjustment of thoughts [32]. This multidimensionality is important for intervention research because interventions may target different regulatory domains or individual components within them, while outcome measures may capture these constructs with substantially different levels of coverage.

A diverse range of non-pharmacological interventions has been used to address self-regulation in autistic children and young people. These include cognitive-behavioural, social-competence, technology-supported, activity-based and classroom-based approaches, which vary substantially in their regulatory targets, delivery methods and opportunities for applying learned skills beyond the intervention setting. This diversity makes it difficult to determine the overall effectiveness of self-regulation-focused interventions or to identify the study, intervention and measurement characteristics associated with stronger effects.

Despite the importance of self-regulation for autistic children and young people, the intervention literature remains difficult to interpret as a coherent evidence base. Previous systematic reviews have generally examined particular intervention approaches or regulatory domains separately [34,35,36,37,38,39,40,41,42,43,44,45,46], limiting comparisons across the broader range of interventions used to support self-regulation in autistic children and young people. These challenges are compounded by substantial conceptual and measurement heterogeneity in self-regulation itself [28]. Though self-regulation encompasses interrelated cognitive, behavioural, and emotional domains, available measures vary considerably in the components they assess, making findings from different instruments difficult to compare directly.

Importantly, evidence of improvement in self-regulation does not necessarily show that intervention benefits extend to children’s everyday functioning. Intervention studies may demonstrate change in a directly targeted regulatory skill or capacity without establishing whether those gains are reflected in social interaction, communication, school activities, adaptive behaviour, mobility or participation. This distinction is particularly important because self-regulation is context-dependent: children’s regulatory performance is shaped by the demands and supports present across home, school and community environments, and may therefore vary according to the context in which it is assessed [47]. Evaluating intervention effectiveness consequently requires assessment beyond the immediate intervention task or setting, including outcomes that capture whether regulatory gains are evident in everyday functioning and across relevant contexts. The central question is therefore not only whether self-regulation can be changed, but whether those changes extend to function, how consistently they do so, and under what conditions functional gains are most likely to occur.

Together, these limitations leave two central gaps. First, there has been no integrated synthesis of randomised controlled trials across the diverse non-pharmacological approaches used to support self-regulation in autistic children and young people. Second, it remains unclear to what extent intervention benefits extend beyond changes in self-regulation to children’s everyday functioning, and which study, participant, intervention or measurement characteristics are associated with variation in these effects.

Accordingly, this systematic review and meta-analysis aimed to identify, characterise and critically evaluate non-pharmacological randomised controlled trials targeting self-regulation in autistic children and young people aged 0–18 years, and to quantify their effects on both self-regulation and separate child functional outcomes. A further aim was to examine whether variation in intervention effects was associated with study design, participant characteristics, intervention characteristics, or the conceptual and measurement characteristics of the outcomes assessed.

The following integrated research questions guided the systematic review and meta-analysis:

1. What are the characteristics of randomised controlled trials of self-regulation-focused interventions for autistic children and young people? Specifically, what study designs, participant characteristics, intervention approaches and techniques, comparators, settings, delivery characteristics, and self-regulation and functional outcome measures have been used?
2. What effects do self-regulation-focused interventions have on self-regulation and on separate child functional outcomes? This includes the magnitude, precision, consistency and between-study variability of pooled effects where sufficient data are available.
3. Which study, participant, measurement and intervention characteristics are associated with variation in intervention effects, and under what conditions do intervention benefits extend to child functioning? For self-regulation, analyses examined comparator type, outcome construct coverage, participant age and intervention duration. For functional outcomes, analyses additionally examined whether effects differed between directly targeted and distal functional outcomes and whether target-skill application or explicit generalisation support was associated with stronger functional effects.
4. How methodologically robust is the evidence for intervention effects on self-regulation and function? This includes the risk of bias of the included trials and the extent to which pooled findings remain stable under prespecified and theoretically justified sensitivity analyses.

## 2. Materials and Methods

The Preferred Reporting Items for Systematic Reviews and Meta-Analyses (PRISMA) statement and checklist were used to establish transparent methodology and reporting [48]. Additionally, A Measurement Tool to Assess Systematic Reviews (AMSTAR-2) [49] informed appraisal and reporting of review methodology. The protocol was registered with PROSPERO (CRD42023482029). The registered protocol initially allowed quasi-experimental studies; the final review was restricted to randomised and cluster-randomised trials to provide a more internally consistent evidence base. Pharmacological and medical interventions were explicitly excluded to retain the non-pharmacological focus. The final review also required at least one separate child functional outcome, whereas function was not specified as a main outcome in the original PROSPERO record. Functional outcome-proximity and intervention-mechanism analyses were subsequently developed as theory-driven exploratory analyses. These deviations and their rationale are summarised in Supplementary Table S12.

### 2.1. Information Sources and Search Strategy

A comprehensive literature search was performed across the following five electronic databases: MEDLINE; PubMed; PsycINFO; ERIC; and CINAHL. See Supplementary Table S1 for search terms per database. All publications up to 24 May 2026 were included.

The theoretical self-regulation literature and related systematic reviews were consulted to identify keywords to include in the search strategy due to the variance in definitions and terms used to describe self-regulation [50,51,52]. All review authors contributed to the development of the search strategy and an academic liaison librarian from the university was consulted.

The search strategy was made up of index terms and keywords categorised into the following two groups: (1) search terms related to autism and (2) search terms related to self-regulation. The search strategy included limits to age and the English language where possible; however, it was not limited by publication date. See Supplementary Table S1 for complete search strategies for each database.

Additional publications were sourced by screening the reference lists of studies deemed eligible for inclusion as well as existing intervention reviews on self-regulation and interrelated terms utilised within the database search strategy. When dissertation articles met eligibility criteria, subsequent searches were completed using Google Scholar to confirm if the study was published in a peer-reviewed journal.

### 2.2. Eligibility Criteria

Studies were required to meet the following eligibility criteria to qualify for inclusion in this review: (1) participants were children and young people, aged 0 to 18 years; (2) participants had a primary formal diagnosis of ASD, including diagnoses of Aspergers Syndrome and Pervasive Development Disorder-Not Otherwise Specified (PDD-NOS), with or without co-occurring conditions; (3) the intervention targeted at least one self-regulation domain (behavioural regulation, cognitive regulation or emotional regulation) as per the operationalised definitions in the Introduction; and (4) the study reported at least one self-regulation and one functional outcome. Studies were required to use an RCT or cluster RCT design.

Studies that had participants over the age of 18 years were included if the mean age of participants was less than or equal to 18 years. Studies were not required to report the criteria used to determine diagnoses of ASD; however, criteria may include editions of the Diagnostic and Statistical Manual of Mental Disorders (i.e., DSM-5 [53]) or International Classification of Diseases, tenth edition (ICD-10) [54].

Studies were excluded if: (1) participants were “at risk” of autism without formal diagnosis, (2) studies used a pilot RCT study design, (3) studies were not published in English, (4) studies were not published in a peer-reviewed journal, or (5) studies investigated components of self-regulation without assessing a functional outcome.

Functional outcomes were defined as measures of the child’s performance, or difficulty in carrying out everyday activities or participating in life situations [55]. Eligibility was guided by the six functioning domains assessed by WHODAS 2.0—cognition, mobility, self-care, getting along, life activities and participation—which correspond to concepts within the ICF framework [56]. Outcomes were eligible when they assessed functioning within at least one of these domains, including communication, social interaction, mobility, self-care, school or daily activities, and participation [55]. Measures limited to symptoms, body functions or impairments, self-regulation itself, caregiver outcomes, family functioning, or parent confidence/empowerment were not considered functional outcomes. Quality-of-life measures were eligible when they assessed the child’s functioning or the impact of health on everyday physical, social, school or other life activities. Measures restricted to general wellbeing, satisfaction or emotional state without an identifiable functional component were not considered functional outcomes.

### 2.3. Selection Process

Covidence [57] was used for study screening and management of records. Before screening commenced, the first and fifth authors independently applied the eligibility criteria to 10 records to calibrate interpretation. The first author screened all titles and abstracts, while the fifth author independently screened a random 50% sample generated within Covidence; the number of records to be screened by the fifth author was agreed before screening commenced. Disagreements, which primarily concerned study design and population eligibility, were resolved through discussion until consensus was reached. Inter-rater agreement was assessed using unweighted Cohen’s κ. The first author subsequently completed full-text screening.

### 2.4. Data Collection Process and Data Items

Two data-extraction tables were developed, piloted and refined by the first, second, third and fourth authors in accordance with guidance from the Cochrane Handbook for Systematic Reviews of Interventions [58]. The first author extracted data from all included studies, and the sixth author reviewed the extracted data for accuracy. Extracted information comprised: (a) participant and study characteristics, including study design, country, study aim, eligibility criteria, participant age, sex, diagnosis, self-regulation and functional outcome measures, and immediate post-intervention and follow-up results; (b) risk of bias; and (c) intervention characteristics, including intervention aim, setting, mode of delivery, recipient, duration, facilitator and relevant training, and intervention content and components. Any discrepancies identified during secondary verification were checked against the primary study reports and corrected before synthesis.

The authors of five eligible RCTs were contacted via email to confirm formal autism diagnoses of participants [59], to clarify participant information [60] or obtain missing outcome data required for synthesis [60,61,62,63]. One study [59] was excluded because a formal autism diagnosis could not be confirmed after attempts to contact the authors.

### 2.5. Assessment of Risk of Bias

The *Cochrane Risk of Bias-2 (RoB-2) Tool for Randomised Trials* [64] was used to assess the risk of bias of individual studies. The RoB-2 is a structured tool containing five domains that assess study trial design, conduct and reporting for risk of bias [64]. The RoB-2 tool uses an algorithm to produce a judgement of “low”, “high” or “some concerns” rating of risk of bias for each domain and study overall [64]. Risk-of-bias judgements were made for the specific immediate post-intervention result contributing to each synthesis. For the three cluster-randomised trials (Kenworthy et al. [65], Lopata et al. [66], and Morgan et al. [67]), the cluster-randomised trial version of RoB-2 was used, including the additional consideration of identification and recruitment in relation to cluster randomisation.

In this systematic review, two independent reviewers completed the RoB-2 checklist for all included studies. Disagreements between raters were resolved by discussion until consensus was gained.

Studies judged to be at high risk of bias were not excluded from the primary meta-analyses. Instead, all eligible studies were retained in the principal models, and sensitivity analyses were conducted to examine whether the pooled findings were robust to exclusion of high-risk-of-bias outcomes. Risk-of-bias judgements were therefore used to evaluate the robustness of the evidence rather than as an a priori exclusion criterion.

### 2.6. Data Synthesis

Study, participant, outcome and intervention characteristics were synthesised descriptively. Interventions were mapped according to their underlying approaches, techniques and practices, informed by existing autism-intervention classification frameworks [68,69].

### 2.7. Meta-Analysis

Outcome framework and selection. The following two linked but analytically distinct meta-analyses were undertaken: one examining intervention effects on self-regulation and one examining effects on separate child functional outcomes. This distinction was central to the review. Improvement in a regulatory construct does not, by itself, demonstrate improvement in everyday functioning; the analyses therefore tested both the targeted regulatory outcome and whether intervention benefits were also evident beyond self-regulation.

Self-regulation was treated as a multidimensional umbrella construct involving the regulation of cognitive processes or thoughts, behaviour, and emotion in the service of goal-directed action. This is aligned with the contemporary measurement synthesis of Janicaud et al. [22], which distinguishes the cognitive, behavioural, and emotional regulation domains and shows that each domain contains multiple related components. Because intervention trials often targeted one domain or one component rather than a comprehensive three-domain construct, eligibility did not require a self-regulation measure to span all domains. Instead, the selected self-regulation outcome was explicitly classified by construct coverage so that component-level, domain-level, and global/multi-domain outcomes could be examined separately. The final review specification and its alignment with contemporary self-regulation measurement are summarised in Supplementary Table S11.

For self-regulation, one outcome was selected per study to preserve statistical independence and prevent studies reporting multiple measures from receiving disproportionate weight. The selected outcome was the measure that most directly represented the self-regulation construct targeted by the intervention. Where more than one eligible measure was available, selection prioritised the prespecified or primary self-regulation outcome and, where this did not resolve selection, the broader validated self-regulation measure rather than a narrower component measure. Construct coverage was coded as component-level, domain-level, or global/multi-domain. This coding was intended to make construct coverage transparent; it did not assume that instruments at different construct-coverage levels were psychometrically interchangeable [22].

One separate child functional outcome was selected per study where available. Where more than one eligible functional outcome was reported, selection prioritised the outcome that most clearly represented the child’s performance of an everyday activity or participation in a life situation and was conceptually distinct from the selected self-regulation outcome. Where more than one outcome met these criteria, preference was given to the broader validated functional measure. Outcome selection was made independently of the magnitude or statistical significance of the reported effect and before classification of functional outcome proximity. Functional outcomes were defined as measures of the child’s performance or difficulty in carrying out everyday activities or participating in life situations. Eligibility was guided by the six functioning domains represented in the World Health Organisation Disability Assessment Schedule 2.0 (WHODAS 2.0) [55] and corresponding activity and participation concepts within the International Classification of Functioning, Disability and Health (ICF) [56]: cognition/communication, mobility, self-care, getting along with others, life activities (including school and daily activities), and participation [55,56]. Measures restricted to symptoms, body functions or impairments, self-regulation itself, caregiver outcomes, family functioning, or parent confidence/empowerment were not classified as child functional outcomes. Quality-of-life measures were eligible where they captured the child’s functioning or the impact of health on everyday physical, social, school or other life activities; measures restricted to general wellbeing or satisfaction were excluded. When the same measure was reported by multiple informants, parent report was selected consistently across studies. Initial outcome selection was undertaken by the first author, with guidance from the second and sixth authors and checking during the meta-analysis/data-audit process rather than independent duplicate selection. The hierarchy was required in 17 studies as follows: 15 required selection among multiple eligible self-regulation outcomes and four among multiple eligible functional outcomes, with Afsharnejad et al. [70] and Lopata et al. [71] requiring a decision for both outcome streams. Study-level selection decisions are documented in Supplementary Table S13.

#### 2.7.1. Functional Outcome Proximity and Intervention-Mechanism Coding

For the functional synthesis, each selected functional outcome was additionally classified according to its proximity to the intervention target. Direct/target-aligned outcomes assessed a functional domain that was explicitly targeted or repeatedly practised by the intervention. Distal/generalisation outcomes assessed function beyond the directly trained or practised intervention target and therefore represented a more stringent test of transfer. Coding was based on intervention content rather than the magnitude or statistical significance of the study effect. The first author completed the initial proximity and mechanism coding, and the sixth author subsequently audited the coding against the published intervention descriptions before the corresponding moderator results were inspected. Discrepancies were resolved through discussion. This was an audit/adjudication process rather than independent duplicate coding; accordingly, an inter-rater coefficient was not calculated.

Three intervention-mechanism variables were defined before the new moderator results were inspected. Target-skill application/rehearsal indicated whether the intervention required repeated enactment or deployment of the targeted regulatory, social, cognitive-functional, or behavioural skill rather than acquisition or capacity training alone. Generalisation support was coded Yes only when the intervention included an explicit mechanism intended to transfer learning beyond the immediate training task or session, such as real-life homework, in vivo assignments, parent or teacher coaching, or home-school transfer procedures. A derived supported application plus generalisation variable was coded Yes only when both target-skill application/rehearsal and explicit generalisation support were present. Insufficiently reported mechanisms were coded Unclear in the audit dataset and treated as missing in the corresponding binary moderator analysis. Broader orientation, naturalistic application, multi-context implementation, key-adult support, explicit functional goals, and direct self-regulation targeting were retained as descriptive coding variables but were not prioritised as formal moderators because of sparse cells or substantial construct overlap. Operational definitions for the functional mechanism variables are provided in Supplementary Table S7; final study-level coding and the inferential status of candidate variables are shown in Supplementary Table S9 and Supplementary Table S10, respectively.

#### 2.7.2. Effect-Size Inputs and Direction

Post-intervention group means, standard deviations and sample sizes were the default effect-size inputs. When these data were unavailable, we retained published standardised effects or appropriate adjusted/model-derived estimates rather than reconstructing unsupported raw values. Five studies required published standardised or adjusted/model-derived inputs for one or both outcome streams. For self-regulation, Kenworthy et al. [65] contributed a published adjusted standardised effect with reported uncertainty; Lopata et al. [66] and Morgan et al. [67] contributed covariate-adjusted post-intervention means, standard deviations and sample sizes; and Lopata et al. [71] and Weiss et al. [72] contributed reported standardised effects (Cohen’s *d*, with 95% confidence intervals where available). For functional outcomes, Lopata et al. [71] and Weiss et al. [72] contributed reported Cohen’s d, while Morgan et al. [67] contributed covariate-adjusted post-intervention means, standard deviations and sample sizes. These inputs were retained because the corresponding unadjusted post-intervention group data required for the common effect-size route were unavailable. All effects were standardised so that positive values favoured intervention. For scales in which higher scores represented poorer outcomes, the mean direction was reversed before analysis while standard deviations and sample sizes were unchanged. The principal meta-analyses used immediate post-intervention outcomes only; follow-up outcomes were extracted and reported descriptively where available but were not pooled with immediate post-intervention effects.

#### 2.7.3. Multi-Arm Trials and Unit of Analysis

For the principal pooled meta-analyses, multi-arm trials were handled by retaining separate eligible intervention comparisons and dividing the shared comparator sample size across comparisons while retaining the comparator mean and standard deviation. This approach was used for the two de Vries et al. [73] comparisons and the two Marzouki et al. [74] comparisons. The principal functional synthesis therefore contained 25 intervention-comparator comparisons from 23 independent studies.

The new categorical functional moderator analyses were conducted at the independent-study level (*k* = 23) because the mechanism and outcome-proximity codes were defined at the intervention-study level and were identical across the relevant multi-arm comparisons. For de Vries et al. [73], the two adaptive cognitive-training intervention groups were combined, and the original mock-control group was used once. For Marzouki et al. [74], the two aquatic intervention groups were combined, and the original treatment-as-usual group was used once. This prevented multi-arm studies from receiving additional weight in the study-level moderator analyses. The functional comparator analysis was rerun using the same 23-study dataset for consistency with the new moderator analyses. The combined study-level inputs used for the de Vries et al. [73] and Marzouki et al. [74] multi-arm trials are documented in Supplementary Table S8. The same combined 23-study dataset was used for functional small-study-effect diagnostics so that each study contributed only one independent effect.

The three cluster-randomised trials were also checked for unit-of-analysis handling. Kenworthy et al. [65] reported a cluster-aware random-effects regression in addition to the primary ANCOVA and reported similar conclusions; the meta-analysis used the published BRIEF^®^ Shift standardised effect from the primary ANCOVA. Morgan et al. [67] used linear mixed models with school modelled as a random effect. Lopata et al. [66] likewise modelled school-level variation and calculated Hedges’ effect sizes for cluster-randomised trials using reported intracluster correlations. These trials were therefore retained, while the Kenworthy effect-size input is stated explicitly as a limitation.

#### 2.7.4. Statistical Analysis

Analyses were conducted in Comprehensive Meta-Analysis, version 3 [75]. Standardised mean differences were expressed as Hedges’ *g* with 95% confidence intervals. Random-effects models were specified a priori because true effects were expected to vary across interventions, participant groups, comparators, settings and measurement approaches [76]. For principal pooled effects, reporting included the standard error, *Z* test, two-tailed *p* value, τ, τ^2^ and 95% prediction interval. Prediction intervals were interpreted as the expected range of true effects in comparable future studies rather than as another test of statistical significance. For descriptive interpretation only, effect sizes around 0.20, 0.50, and 0.80 were considered small, moderate, and large, respectively [77]; interpretation prioritised confidence intervals, prediction intervals, and the clinical meaning of the outcome. The principal analyses used inverse-variance random-effects models with the DerSimonian–Laird method-of-moments estimator for between-study variance and conventional 95% confidence intervals. As a small-sample sensitivity analysis, Hartung–Knapp inference was additionally applied to the two principal pooled effects using the same study-level Hedges’ g estimates, sampling variances and between-study variance estimates [78]. Moderator analyses were treated as exploratory, particularly where subgroup sizes were small or unequal; non-significant moderator tests were not interpreted as evidence that an association was absent.

Categorical moderators were examined using mixed-effects subgroup models, with random effects within subgroups and a fixed-effect *Q*_β_ test of differences between subgroups. Subgroup reporting included *k*, Hedges’ *g*, 95% CI, τ (and τ^2^ in the Supplementary Material), subgroup prediction intervals where estimable, and the *Q*_β_ test. Comparator type was examined for self-regulation and function, and self-regulation outcome construct coverage was examined as a construct-specific moderator. For the independent-study functional analyses, functional outcome proximity was the primary new moderator. Supported application plus generalisation was examined as the principal intervention-mechanism moderator, with target-skill application/rehearsal and explicit generalisation support examined as secondary mechanism moderators. Broad intervention type was retained as an exploratory contextual moderator. Mean participant age and intervention duration in weeks were examined as separate univariable random-effects meta-regressions for both outcomes. Full categorical moderator statistics, including subgroup τ^2^ estimates and prediction intervals where estimable, are reported in Supplementary Table S4.

The functional mechanism analyses were theory-driven exploratory analyses rather than protocol-prespecified tests. However, coding definitions, intervention classifications, and the analysis hierarchy were finalised before the corresponding moderator results were inspected. Because these are study-level moderators, they were interpreted as associations and not as evidence that a specific intervention ingredient causally produced functional change.

#### 2.7.5. Sensitivity Analyses

All eligible effects were included in the primary models. For self-regulation, five independent sensitivity analyses tested methodological robustness, comparator/design robustness, construct-definition robustness, and analytic-input robustness. For functional outcomes, the prespecified sensitivity analysis excluded high-risk-of-bias outcomes. A further targeted sensitivity analysis examined the strongest new functional moderator, outcome proximity, after restricting the study-level dataset to trials with active/attention comparators. This analysis tested whether the direct-versus-distal contrast persisted when comparator stringency was more closely held constant. The complete sensitivity-analysis results are reported in Supplementary Table S3. The self-regulation comparator/design sensitivity excluded comparisons flagged as active-comparator or component-add-on designs. This is distinct from the targeted functional proximity sensitivity, which restricted the independent-study dataset to active/attention-comparator trials.

#### 2.7.6. Small-Study Effects

Small-study effects were examined using funnel plots, Begg and Mazumdar’s rank correlation [79], Egger’s regression, Rosenthal’s fail-safe N [80], and Duval and Tweedie’s trim-and-fill procedure [81]. For self-regulation, diagnostics used the 27 intervention-comparator effects in the principal synthesis. For functional outcomes, diagnostics used 23 independent study-level effects, combining the intervention arms in de Vries et al. [73] and Marzouki et al. [74] so that each study contributed once. These analyses were interpreted as diagnostics of small-study asymmetry rather than proof of publication bias; fail-safe N was retained descriptively only, and trim-and-fill was treated as an assumption-dependent sensitivity model. Full diagnostics are reported in Supplementary Table S6, with the self-regulation funnel plot in Supplementary Figure S1.

## 3. Results

### 3.1. Study Selection

In total, 5284 records were retrieved from the five electronic databases searched and imported into Covidence [57]. Before title and abstract screening, 2019 duplicate records were removed electronically by Covidence [57] (n = 1994) and manually (n = 25). The first author independently assessed the remaining 3265 title and abstract records, and the fifth author screened a random 50% verification sample. For the 1691 records screened by both reviewers, there were 36 discrepant decisions, primarily concerning study design and population eligibility. Observed agreement was 97.9%, with unweighted Cohen’s κ = 0.812 (95% CI 0.752–0.872) [82].

**Figure 1:**
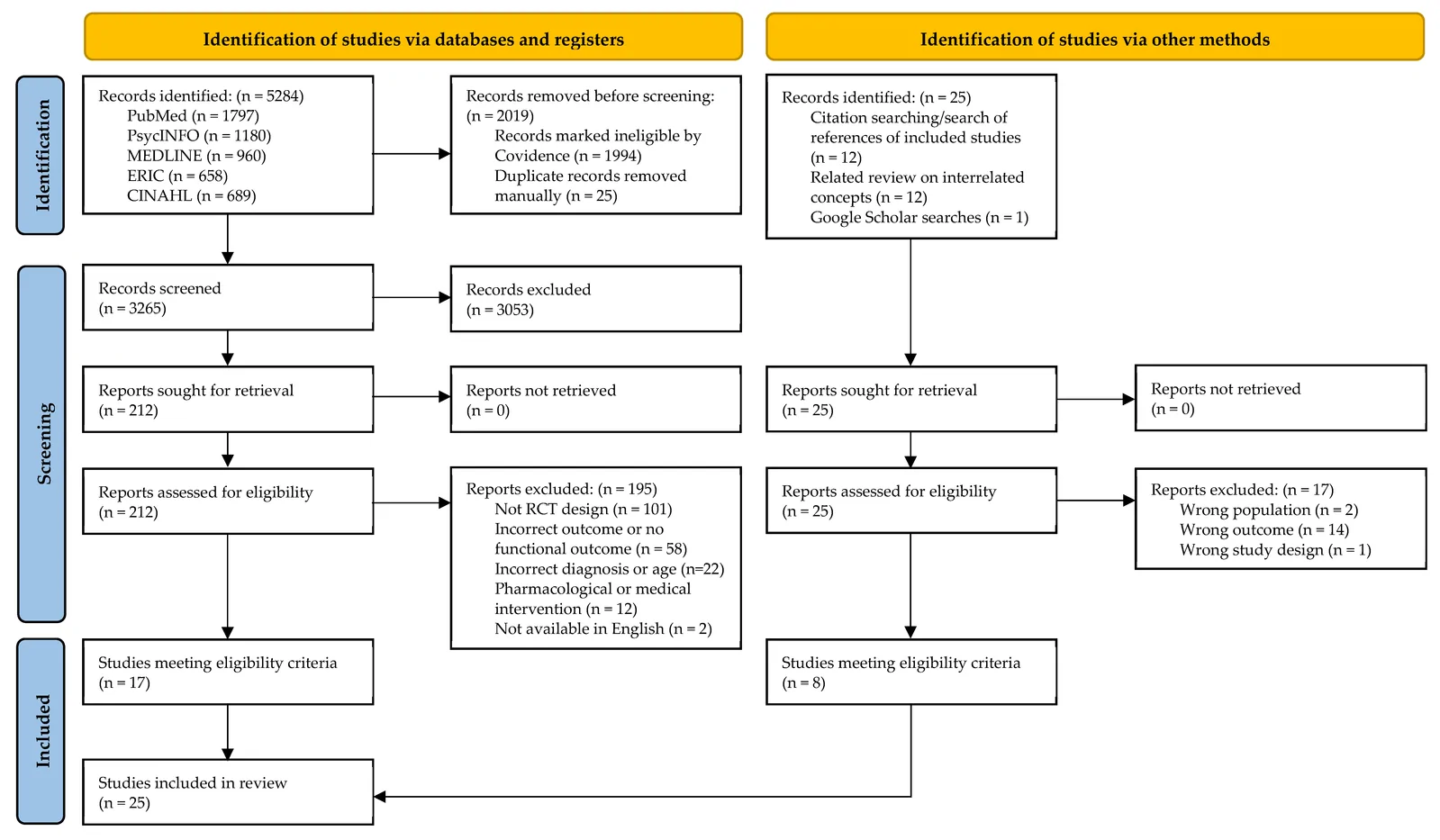
PRISMA flow diagram.

A total of 212 full-text articles were retrieved and assessed for eligibility. In addition, 25 full-text articles were identified from reference searches of included studies and related systematic reviews as well as *Google Scholar* searches. Reviewers identified 17 studies that met the inclusion criteria, along with eight studies identified via other methods. Overall, a total of 25 studies were included for data extraction. See Figure 1 for the PRISMA flow diagram.

### 3.2. Study Characteristics

The characteristics of the 25 included studies are described in detail within Table 1. Twelve studies were conducted in the United States of America, four studies in Australia and two in the Netherlands. The remaining studies were undertaken in the United Kingdom, Canada, Italy, Germany, North Africa, China and Malaysia. Studies were published between 2008 and 2025.

All 25 included studies used randomised controlled designs, including three cluster-randomised trials conducted in school settings. Across the studies, 1655 participants were randomised (mean study sample size = 66.2, *SD* = 41.1), with study sample sizes ranging from 23 to 208 participants.

Comparator conditions included waitlist/no-treatment, treatment-as-usual and active/attention control conditions. The de Vries et al. [73] and Marzouki et al. [74] trials each included two intervention arms and one comparator arm.

### 3.3. Participants

Across the included studies, mean participant age ranged from approximately 4.4 to 17.9 years, with individual participant ages spanning approximately 3 to 19 years. Participants were predominantly male. Most studies required a formal clinical autism diagnosis, although Morgan et al. [67] permitted either a clinical or educational diagnosis. Diagnostic criteria and confirmation procedures varied across trials, with DSM-based criteria used most commonly and ICD-10 [54] used in one study. Many studies also imposed cognitive or language eligibility thresholds, most commonly requiring IQ scores above approximately 70.

### 3.4. Outcome Measures

All studies completed pre- and post-intervention outcome measurements, with a subset also reporting follow-up assessments ranging from approximately one to five months after intervention. Beaumont et al. [86] completed follow-up assessments at two time points post-intervention (i.e., six weeks and five months).

#### 3.4.1. Measures of Self-Regulation

Self-regulation was assessed using a wide range of outcome measures spanning the cognitive, behavioural and emotional regulation domains. The emotional and cognitive regulation domains were most frequently represented, while fewer studies assessed the behavioural regulation domain explicitly. Measures varied substantially in construct coverage: some assessed a single regulatory component, whereas others assessed a broader domain or multiple self-regulation domains. This heterogeneity in construct coverage informed the component-level, domain-level and global/multi-domain classifications used in the meta-analysis.

Assessment methods also varied, including direct child tasks, parent- and teacher-report questionnaires, clinician ratings and observational measures. Some instruments assessed self-regulation in everyday contexts, whereas others focused more narrowly on regulatory performance under structured task conditions.

#### 3.4.2. Measures of Function

Functional outcomes represented a range of everyday life domains, including social functioning, communication, adaptive behaviour, school participation and performance, mobility and motor activity performance. Measures included teacher-, clinician- and self-report instruments as well as direct and observational assessments. For the meta-analysis, functional outcomes were distinguished from self-regulation outcomes and were required to assess the child’s performance or difficulty in carrying out activities or participating in identifiable life situations.

### 3.5. Interventions

The included trials evaluated a diverse range of non-pharmacological self-regulation-focused interventions. These included cognitive-behavioural and psychosocial approaches, technology-supported interventions, activity- and movement-based interventions, social-cognitive programmes, classroom-based approaches and other structured skills-based interventions. Intervention delivery varied across individual, group, home, clinic, school and online formats, and facilitators included clinicians, teachers, parents and other trained interventionists. Table 2 provides detailed descriptions of intervention content, dosage, delivery and implementation characteristics.

### 3.6. Risk of Bias

Risk-of-bias judgements varied across the included trials, with concerns arising most frequently from outcome measurement and missing outcome data. In behavioural intervention trials, inability to blind participants or interventionists was considered only insofar as awareness of allocation could plausibly produce deviations from the intended intervention or influence measurement of the selected outcome; lack of blinding alone was not treated as establishing high risk of bias. Outcome-specific judgements were used for the meta-analytic sensitivity analyses because risk of bias could differ according to the result selected within the same study. High-risk outcomes were retained in the principal analyses and excluded in sensitivity analyses to assess robustness. The principal findings remained stable after these exclusions, as reported below. See Table 1 for details.

### 3.7. Meta-Analysis 

#### 3.7.1. Principal Pooled Effects

**Figure 2:**
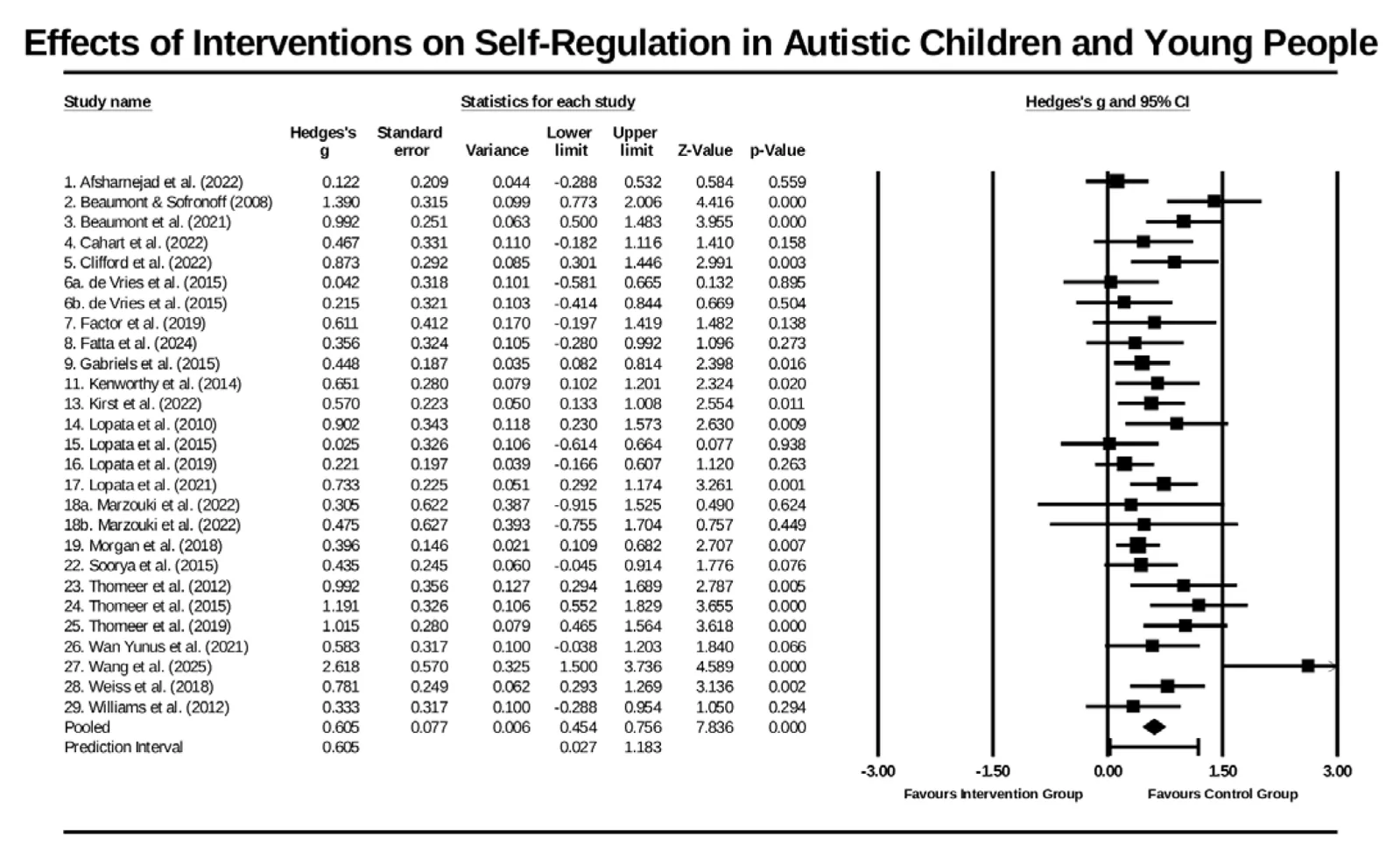
Random-effects meta-analysis of intervention effects on self-regulation in autistic children and young people. The diamond shape represents the pooled effect of interventions on self-regulation.

The self-regulation meta-analysis included 27 intervention-comparator effects from 25 independent studies, see Figure 2. Under the random-effects model, interventions produced a clear positive effect on self-regulation (Hedges’ *g* = 0.605, *SE* = 0.077, 95% CI 0.454 to 0.756, *Z* = 7.836, and *p* < .001; *τ* = 0.270, *τ^2^* = 0.073). The 95% prediction interval ranged from 0.027 to 1.183. Thus, although the magnitude of the true effect is expected to vary, the prediction interval remained just above zero across comparable future settings.

**Figure 3:**
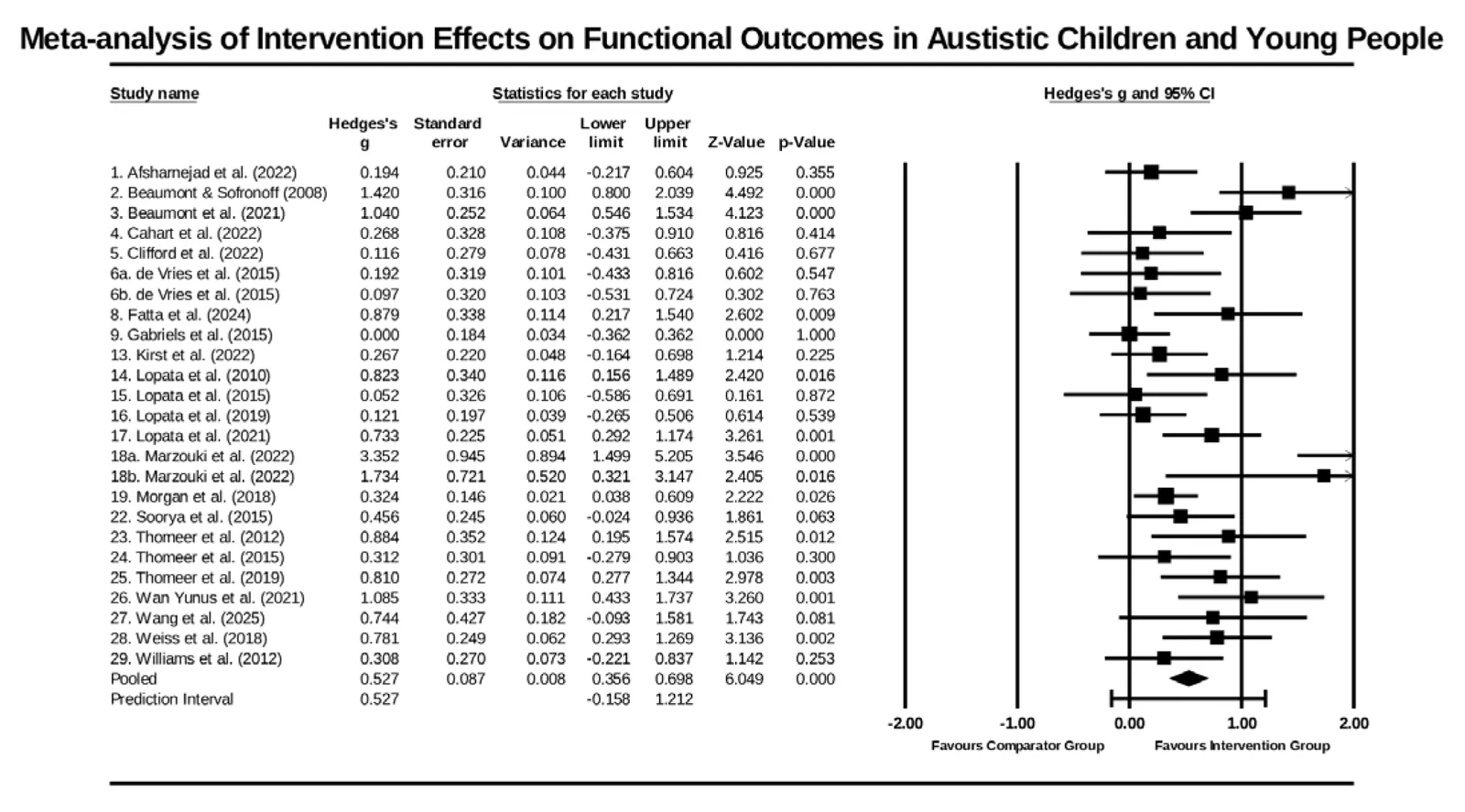
Random-effects meta-analysis of intervention effects on separate child functional outcomes.

The functional-outcome meta-analysis included 25 intervention-comparator effects from 23 independent studies, see Figure 3. Under the random-effects model, interventions produced a positive average effect on separate child functional outcomes (Hedges’ *g* = 0.527, *SE* = 0.087, 95% CI 0.356 to 0.698, *Z* = 6.049, and *p* < .001; *τ* = 0.320, *τ^2^* = 0.102). The 95% prediction interval ranged from −0.158 to 1.212. Intervention benefits therefore extended beyond self-regulation at the pooled level, but true functional effects were more variable across contexts than self-regulation effects. Full statistical reporting for both principal pooled models is provided in Supplementary Table S2. Hartung–Knapp sensitivity analyses yielded the same substantive conclusions with modestly wider confidence intervals as follows: self-regulation, *g* = 0.605, Hartung–Knapp 95% CI 0.435 to 0.775, *t*(26) = 7.31, and *p* < .001; functional outcomes, *g* = 0.527, Hartung–Knapp 95% CI 0.328 to 0.725, *t*(24) = 5.48, and *p* < .001.

#### 3.7.2. Moderator and Meta-Regression Analyses

Comparator type moderated both outcome streams. For self-regulation, comparator type significantly moderated effects (*Q*_β_ = 12.234, *df* = 2, and *p* = .002): effects were largest against waitlist/no treatment (*k* = 9; *g* = 0.878, and 95% CI 0.682 to 1.074; *τ* = 0), followed by treatment as usual (*k* = 5; *g* = 0.747, 95% CI 0.021 to 1.474; and *τ* = 0.685), and were smaller but remained positive against active/attention comparators (*k* = 13; *g* = 0.444, 95% CI 0.297 to 0.590; and *τ* = 0.108). In the independent-study functional dataset, comparator type also significantly moderated effects (*Q*_β_ = 7.167, *df* = 2, and *p* = .028). Functional effects were smallest against active/attention comparators (*k* = 11; *g* = 0.335, 95% CI 0.145 to 0.525; and *τ* = 0.216), and larger against treatment as usual (*k* = 4; *g* = 0.727, 95% CI −0.021 to 1.476; and *τ* = 0.660) and waitlist/no treatment (*k* = 8; *g* = 0.739, 95% CI 0.502 to 0.977; and *τ* = 0.179). See Table 3 for key moderator results. Full comparator subgroup statistics are reported in Supplementary Table S4; the self-regulation comparator forest plot is shown in Supplementary Figure S2.

Functional outcome proximity produced the clearest new moderator signal. Effects were substantially larger when the selected functional outcome was directly aligned with the intervention target (*k* = 13; *g* = 0.761, 95% CI 0.496 to 1.027; and *τ* = 0.382) than when the functional outcome was distal to the trained or practised target (*k* = 10; *g* = 0.250, 95% CI 0.108 to 0.393; and *τ* = 0.000). The between-group difference was significant (*Q*_β_ = 11.034, *p* < .001). The 95% prediction interval for direct/target-aligned outcomes was −0.131 to 1.654, indicating substantial variation in the magnitude of direct functional effects. In contrast, no between-study variance was estimated for the distal subgroup. Importantly, the distal pooled effect remained positive, indicating that benefits were not confined to functional behaviours directly targeted by intervention.

The more specific intervention-mechanism analyses did not identify statistically significant moderator effects. Effects were numerically larger when interventions combined target-skill application with explicit generalisation support (Yes: *k* = 13, *g* = 0.578, and 95% CI 0.365 to 0.792; τ = 0.290; No: *k* = 10, *g* = 0.409, and 95% CI 0.138 to 0.681; τ = 0.324), but the between-group difference was not significant (*Q*_β_ = 0.918, *df* = 1, and *p* = .338). Target-skill application/rehearsal alone was also not a statistically significant moderator (Yes: *k* = 15, *g* = 0.578, and 95% CI 0.385 to 0.770; τ = 0.278; No: *k* = 8, *g* = 0.354, and 95% CI 0.031 to 0.678; τ = 0.345; *Q*_β_ = 1.353, *df* = 1, and *p* = .245). After excluding the one study with unclear coding, explicit generalisation support was likewise not statistically significant (Yes: *k* = 13, *g* = 0.578, and 95% CI 0.365 to 0.792; τ = 0.290; No: *k* = 9, *g* = 0.430, and 95% CI 0.128 to 0.731; τ = 0.352; *Q*_β_ = 0.622, *df* = 1, and *p* = .430). Broad intervention type was not a statistically significant moderator (*Q*_β_ = 0.487, *df* = 2, and *p* = .784). Given the modest and unequal subgroup sizes, these non-significant tests should not be interpreted as demonstrating absence of an association.

Self-regulation construct coverage did not alter the intervention effect (*Q*_β_ = 0.883, *df* = 2, and *p* = .643). Pooled effects were positive at each level as follows: component-level (*k* = 6; *g* = 0.553, 95% CI 0.337 to 0.768; and *τ* = 0.036), domain-level (*k* = 9; *g* = 0.719, 95% CI 0.438 to 1.000; and *τ* = 0.282), and global/multi-domain (*k* = 12; *g* = 0.583, 95% CI 0.317 to 0.849; and *τ* = 0.357). The null moderator result indicates that pooled intervention effects were not systematically larger at one construct-coverage level; it should not be interpreted as evidence that measures with different construct coverage are equivalent.

No statistically significant linear associations were detected for mean age or intervention duration. Mean participant age was not associated with self-regulation effect size (β = −0.027, 95% CI −0.088 to 0.035, and *p* = .391; residual τ = 0.284) or functional effect size (β = −0.026, 95% CI −0.094 to 0.042, and *p* = .460; residual τ = 0.333). Intervention duration was also unrelated to self-regulation effects (β = 0.002 per week, 95% CI −0.015 to 0.020, and *p* = .788; residual τ = 0.300) or functional effects (β = 0.007 per week, 95% CI −0.040 to 0.054, and *p* = .770; residual τ = 0.360). Full construct-coverage subgroup statistics and the corresponding forest plot are presented in Supplementary Table S4 and Figure S3. Full meta-regression results are reported in Supplementary Table S5; the self-regulation mean-age and duration models are illustrated in Supplementary Figures S4 and S5, and the functional mean-age model in Supplementary Figure S6.

#### 3.7.3. Sensitivity Analyses

The principal findings were not dependent on any single operational or methodological decision. For self-regulation, excluding seven high-risk-of-bias comparisons retained 20 comparisons and yielded *g* = 0.538 (95% CI 0.400 to 0.677; τ = 0.153). A separate self-regulation sensitivity analysis excluding comparisons flagged as active-comparator or component-add-on designs retained 15 comparisons and produced *g* = 0.818 (95% CI 0.581 to 1.055; τ = 0.323). Excluding component-only outcomes retained 21 comparisons (*g* = 0.624, 95% CI 0.434 to 0.813; τ = 0.318), excluding hybrid self-regulation/function outcomes retained 17 comparisons (*g* = 0.544, 95% CI 0.368 to 0.720; τ = 0.208), and excluding effects based on non-standard or model-derived input routes retained 22 comparisons (*g* = 0.635, 95% CI 0.444 to 0.826; τ = 0.324). Every self-regulation sensitivity estimate remained positive and statistically significant.

For functional outcomes, exclusion of five high-risk-of-bias comparisons left 20 comparisons. The pooled functional effect remained positive and statistically significant (*g* = 0.488, 95% CI 0.306 to 0.670; *τ* = 0.297), demonstrating that the functional finding was not driven by the high-risk studies.

The outcome-proximity finding was then stress-tested within the 11 studies using active/attention comparators. The same substantive gradient remained: directly target-aligned outcomes showed a moderate-to-large effect (*k* = 4; *g* = 0.659, 95% CI 0.220 to 1.099; and τ = 0.367), whereas distal/generalisation outcomes showed a smaller positive effect (*k* = 7; *g* = 0.196, 95% CI 0.038 to 0.355; and τ = 0.000). The between-group test attenuated to *Q*_β_ = 3.770, *p* = .052. Thus, restricting the analysis to active/attention comparators reduced certainty that outcome proximity operates independently of comparator design, but it did not reverse the marked separation in the direct and distal point estimates. Full sensitivity-analysis results are presented in Supplementary Table S3.

#### 3.7.4. Small-Study Effects

Small-study diagnostics did not overturn the self-regulation finding. Begg and Mazumdar’s rank correlation was not significant (Kendall’s *τ-b* = 0.182, two-tailed *p* = .182), while Egger’s intercept was 1.631 (95% CI −0.080 to 3.343; two-tailed *p* = .061). The classic fail-safe N was 834. Under the trim-and-fill model, four studies were imputed and the pooled estimate decreased from *g* = 0.605 to *g* = 0.497 (95% CI 0.325 to 0.669). The adjusted estimate therefore remained clearly positive. Full numerical diagnostics are provided in Supplementary Table S6, with the funnel plot shown in Supplementary Figure S1.

Corresponding diagnostics for the 23 independent study-level functional effects indicated funnel asymmetry. Begg–Mazumdar rank correlation was significant (Kendall’s τ-b = 0.399, two-tailed *p* = .007), as was Egger’s regression (intercept = 3.403, 95% CI 1.356 to 5.451, and *p* = .002). Duval–Tweedie trim-and-fill imputed six studies on the left side of the funnel and reduced the pooled functional estimate to *g* = 0.357 (95% CI 0.165 to 0.549); the classic fail-safe N was 519 and is reported descriptively only. These diagnostics indicate small-study asymmetry but do not establish publication bias. Under the trim-and-fill model, the adjusted functional estimate remained positive and statistically distinct from zero. Full diagnostics for both outcome streams are reported in Supplementary Table S6.

## 4. Discussion

This systematic review and meta-analysis synthesised randomised controlled trials of non-pharmacological interventions targeting self-regulation in autistic children and young people. Across a diverse evidence base, interventions varied substantially in theoretical approach, delivery format, regulatory target and outcome measurement. Despite this heterogeneity, the meta-analysis identified clear positive average effects on self-regulation and on separate child functional outcomes. The findings therefore extend the descriptive systematic-review literature by showing not only that self-regulation-focused interventions can produce measurable regulatory change, but also that these benefits can extend into child functioning.

The systematic review itself identified substantial diversity in how self-regulation was addressed. Interventions drew on cognitive-behavioural, psychosocial, technology-supported, activity- and movement-based, social-cognitive and classroom-based approaches and were delivered across individual, group, home, clinic, school and online formats. This diversity is consistent with the broader autism-intervention literature, in which intervention approaches are organised around markedly different theoretical principles and techniques [68,69]. Rather than pointing to a single dominant model of self-regulation intervention, the present review shows that regulatory change is being pursued through multiple pathways, involving children directly as well as parents, teachers and other interventionists. This breadth is a strength of the evidence base, but it also complicates attempts to determine which intervention characteristics are responsible for stronger outcomes.

A second prominent finding was the conceptual and measurement heterogeneity surrounding self-regulation. Trials assessed the cognitive, behavioural and emotional regulation domains using measures that varied from relatively narrow component-level tasks to broader domain and multi-domain instruments. This reflects a wider problem in the self-regulation literature. Contemporary measurement work has shown substantial variation in which components are represented by available self-regulation measures and that no single instrument captures the construct comprehensively [22]. The issue is not merely psychometric. Self-regulation is also context-sensitive: developmental accounts emphasise that regulatory performance is shaped by environmental demands and can vary across home, school and other everyday contexts [18]. Consequently, intervention studies relying primarily on decontextualised or narrowly targeted regulatory measures may demonstrate acquisition of a regulatory skill without establishing how consistently that skill is available or used in everyday situations.

The review therefore also reinforces the importance of distinguishing regulatory outcomes from functioning. Functional outcomes in the included trials encompassed social interaction, communication, adaptive behaviour, school-related activities, mobility and participation, but their relationship to intervention content varied considerably. Some assessed behaviours that were explicitly taught or repeatedly practised, whereas others required application beyond the immediate intervention target. This distinction is important because effective self-regulation ultimately involves adapting cognition, emotion and behaviour to changing situational demands, rather than demonstrating regulatory capacity only under the conditions in which it was trained [18]. Methodological limitations further qualify interpretation of the evidence: risk-of-bias concerns arose particularly in relation to outcome measurement, missing outcome data and the practical difficulty of blinding participants, interventionists and assessors in behavioural trials. Taken together, the systematic-review findings show the intervention literature that is broad, but conceptually heterogeneous, context-sensitive and methodologically uneven. These characteristics provide the necessary context for interpreting the pooled effects and, particularly, the extent to which benefits extend beyond proximal regulatory change into child functioning.

The central finding is not simply that interventions improve both self-regulation and child functioning. Contemporary syntheses have established that emotion-regulation interventions can improve regulatory outcomes in autistic children and adolescents [45] and a 2026 network meta-analysis further showed that several intervention classes produce meaningful emotion-regulation effects, with effectiveness varying across intervention and contextual characteristics [179]. That literature primarily establishes whether regulatory outcomes change. The present review extends that evidence by asking what happens after the proximal regulatory target: functional effects were substantially larger when the measured functional behaviour was directly targeted or repeatedly practised by the intervention (*g* = 0.761) than when the outcome represented function beyond the immediate intervention target (*g* = 0.250). The principal advance is therefore identifying a functional proximity gradient rather than another demonstration of proximal efficacy.

The distal effect is especially important. A pooled effect of *g* = 0.250 remained positive when the selected functional outcome was not directly trained or practised, with τ^2^ estimated as zero in this subgroup; given the small number of studies, this estimate should not be interpreted as proof of homogeneity. This indicates that intervention benefits can extend beyond behaviours that closely mirror intervention content. The finding speaks directly to the transfer problem identified across autism intervention research. Gates et al. [180], for example, found that large self-reported gains after group social-skills interventions were attributable to increased social knowledge rather than self-reported enactment, while teacher-reported effects were not significant. Wyman and Claro [181] similarly found improved social knowledge after a school-based PEERS^®^ programme but greater difficulty among autistic participants in applying new skills outside the programme. More recently, Cheng et al. [182] concluded that PEERS^®^ can improve both social knowledge and skill application, while Nejati et al. [183] explicitly foregrounded transferability across function, implementation, ecology, level, and durability. Against that background, the present distal estimate quantitatively isolates the more demanding question of whether functional benefits remain when the measured outcome extends beyond what was directly trained.

At the same time, the size of the direct-versus-distal contrast shows that generalisation cannot be assumed. This is consistent with reviews concluding that social-skills interventions can improve social competence but remain limited in generalisability and scope [184], and with the 2024 transfer analysis showing that transfer is shaped by intervention context, intensity, tools, age, and other design features rather than occurring automatically [183]. It is also consistent with the executive-function literature: interventions can improve working memory, flexibility, and related behavioural outcomes, yet evidence for broad everyday transfer remains less developed than evidence for change in the trained executive process itself [185]. The recent review of emotion regulation in autistic adolescents likewise highlights heterogeneity in measurement and context [186]. The current synthesis operationalises that ecological problem directly: target-aligned outcomes are closer to what was taught, rehearsed, prompted, or reinforced, whereas distal outcomes require transfer across tasks, settings, social partners, and environmental demands.

Comparator design remains central to this interpretation. In the independent-study functional dataset, effects were smaller against active/attention comparators than against waitlist/no-treatment controls, and comparator type remained a significant moderator. Distal outcomes were also disproportionately represented in active-comparator trials. The targeted active-comparator sensitivity analysis therefore provides an important stress test. When the proximity analysis was restricted to 11 active-comparator studies, the effect-size contrast remained sizeable (*g* = 0.659 for direct outcomes versus *g* = 0.196 for distal outcomes), although the between-group test attenuated to *p* = .052. The loss of conventional statistical significance should not be interpreted as reversal of the proximity pattern: the point-estimate separation remained large, but precision decreased markedly after restriction to four direct and seven distal studies. The appropriate conclusion is that comparator design contributes to the observed proximity gradient but does not fully account for its magnitude.

The intervention-mechanism analyses add a second important insight. Neither target-skill application, explicit generalisation support, nor their combination significantly moderated functional effects. This argues against a simple account in which functional transfer can be explained by the presence of rehearsal, homework, parent/teacher transfer support, or another single coded ingredient. Importantly, this does not conflict with evidence that programmes such as PEERS^®^ can support skill application [182], nor does it imply that generalisation procedures are unimportant. Rather, a binary study-level code for whether a component is present cannot capture its dose, quality, fidelity, timing, or fit with the child’s environments. The context-dependence reported in contemporary emotion-regulation syntheses [179,186] is compatible with the present result: successful transfer may depend on interactions among practice quality, ecological opportunity, adult and peer responses, environmental contingencies and the functional relevance of the trained skill, rather than on any single intervention ingredient in isolation.

The prediction intervals reinforce this interpretation at the broader outcome level. For self-regulation, the 95% prediction interval remained above zero, indicating that a favourable true effect would be expected across a broad range of comparable future settings. For the overall functional synthesis, the prediction interval crossed zero, indicating greater contextual variation in true effects without overturning the positive pooled average effect. The proximity analysis refines that observation: direct effects are larger on average but heterogeneous, whereas distal effects are smaller and had τ^2^ estimated as zero in the present dataset, an estimate that should not be interpreted as proof of homogeneity. This distinction helps reconcile the growing evidence that emotion-regulation interventions are efficacious [45,178] with persistent concerns about ecological validity and everyday translation [179,185]. The challenge for intervention science is therefore not simply to maximise proximal change, but to understand the conditions under which that change becomes usable and sustained in everyday life.

Self-regulation outcome construct coverage did not explain intervention effects. Positive effects were evident for component-level, domain-level, and global/multi-domain measures, and the between-group test was non-significant. This finding aligns with the purpose of construct coverage coding but should not be overextended. Janicaud et al. [22] showed substantial variation in the components covered by self-regulation instruments and found that no single measure fully captured the construct. The present result therefore supports robustness of the intervention signal across different levels of construct coverage; it does not establish measurement equivalence. This distinction also strengthens the case for future trials to report precisely which self-regulation domains and constituent components are targeted and measured, rather than using the umbrella label “self-regulation” without specifying its operational content.

The measurement implications also extend beyond construct coverage. Janicaud et al. [22] argued for assessment that captures self-regulation across real-life contexts, including ecologically intensive approaches. That recommendation aligns with the present functional findings: if self-regulation is dynamic and context-sensitive, then a trial architecture limited to decontextualised regulatory measures risks overestimating how usable change becomes in everyday life. Pairing proximal self-regulation with target-aligned and distal functional outcomes would provide a more coherent test of both acquisition and transfer.

The sensitivity analyses materially strengthen confidence in the principal conclusions. The self-regulation effect remained positive after removing high-risk-of-bias outcomes, component-only measures, hybrid self-regulation/function outcomes, and studies requiring non-standard or model-derived effect-size inputs. Functional effects likewise remained positive after excluding high-risk outcomes. The active-comparator proximity sensitivity did not reproduce the full-sample between-group *p* value but retained the same marked difference in effect magnitude and a significant positive distal effect. This is appropriately interpreted as attenuation of the evidence, not reversal of the pattern.

The small-study analyses refine rather than overturn the principal findings. For self-regulation, the rank-correlation test was non-significant, and Egger’s test was close to, but did not cross, the conventional two-tailed threshold. The trim-and-fill procedure imputed four studies and reduced the pooled estimate from 0.605 to 0.497, which remained clearly positive. For functional outcomes, Begg–Mazumdar and Egger tests indicated funnel asymmetry. Under the trim-and-fill model, six studies were imputed and the pooled estimate decreased from *g* = 0.527 to *g* = 0.357 (95% CI 0.165 to 0.549). Funnel asymmetry does not establish publication bias and trim-and-fill is assumption-dependent; nevertheless, the adjusted functional estimate remained positive and statistically distinct from zero. The diagnostics therefore qualify the likely magnitude of the functional effect rather than eliminate evidence for a positive average functional effect.

### 4.1. Implications for Intervention Research and Outcome Measurement

The findings support a more deliberate outcome architecture for future trials. Demonstrating change in the targeted regulatory construct is necessary but insufficient. Trials should distinguish at least two levels of functional evidence as follows: a target-aligned functional outcome showing whether the intervention changes the everyday behaviour it directly addresses, and, where theoretically justified, a distal functional outcome showing whether benefits generalise beyond what was explicitly trained. This recommendation directly addresses concerns in the social-skills literature that knowledge gains may exceed enacted behaviour [180,181] and the contemporary call for ecologically valid emotion-regulation measurement [186]. Ideally, trials would assess proximal self-regulation, target-aligned function, and distal function within the same design and formally test the pathways linking them. This would allow the field to move from parallel evidence of change toward direct tests of mediation and generalisation.

This distinction also has implications for how intervention success is described. Large effects on outcomes that closely reproduce intervention content should not automatically be interpreted as broad functional transformation. Conversely, a smaller effect on a distal outcome may represent a more demanding and clinically meaningful form of transfer. Contemporary intervention reviews increasingly emphasise context and application rather than efficacy alone [179,182], and the present results provide a quantitative framework for doing so. Reporting both effect magnitude and outcome proximity would make claims about intervention success more transparent and prevent proximal learning from being conflated with generalised everyday function.

The comparator findings have equally direct implications for trial design. Waitlist and treatment-as-usual comparisons remain valuable for estimating pragmatic added benefit, but active comparators are essential when the objective is to isolate intervention-specific effects. Future trials should state clearly which estimand they are designed to address and select comparator conditions accordingly. The present active-comparator sensitivity also illustrates why mechanistic moderator findings should be stress-tested against design features before being interpreted as properties of the intervention itself.

### 4.2. Limitations

Several limitations of the systematic review should also be considered. Eligibility was restricted to peer-reviewed randomised controlled trials published in English, which may have excluded relevant evidence from other study designs or languages. Embase, Scopus and Web of Science were not searched, and the peer-reviewed English-language restriction may have introduced language- or publication-related selection. Considerable heterogeneity in intervention approaches and outcome measurement complicated direct comparison across studies, and several intervention and transfer-related characteristics were incompletely reported. Some functional outcomes were unavailable in a form suitable for quantitative synthesis, meaning that not every eligible functional outcome could contribute to the pooled estimate. The age criterion was based on mean study age, so a small number of individual participants were older than 18 years; this defines a boundary on the population to which the findings should be generalised.

The evidence base remains modest for moderator and meta-regression analyses. The functional mechanism analyses were theory-driven, and coding was finalised before those results were inspected, but they were not prespecified in the registered protocol and should therefore be considered exploratory. All moderator analyses operate at the study level: they cannot establish that the same children improved in self-regulation and function, nor can they establish mediation or causal active ingredients. “Direct” and “distal” describe conceptual proximity of the selected functional outcome to intervention content, not an empirically observed causal distance. Outcome proximity was associated with comparator design, and the active-comparator sensitivity contained only four direct and seven distal studies. Functional outcomes were deliberately broad across life domains, so the results do not imply equal effects within every domain. Mechanism coding was constrained by published intervention descriptions, and some transfer-related features could not be classified confidently. Some studies required published standardised effects or adjusted/model-derived inputs. Small-study diagnostics identified a stronger asymmetry signal for functional outcomes than for self-regulation, but these methods cannot establish the cause of funnel asymmetry and trim-and-fill is an assumption-dependent sensitivity model. A formal certainty-of-evidence framework such as GRADE was not prespecified or undertaken; conclusions are therefore interpreted in relation to outcome-specific risk of bias, between-study variability, prediction intervals, sensitivity analyses and small-study-effect diagnostics.

In summary, interventions produced beneficial average effects on both self-regulation and separate child functional outcomes, but functional improvement was strongly graded by proximity to the intervention target. Directly targeted function improved substantially more than distal function, yet the positive distal effect indicates that benefits can extend beyond what is explicitly trained. This connects contemporary evidence of intervention efficacy in emotion regulation [45,179] with the persistent problem of translating acquired knowledge or skills into enacted behaviour across settings [180,181,183]. The contrast attenuated but remained sizeable under active-comparator restriction, indicating that both outcome proximity and trial design shape observed functional effects. Specifically coded intervention features—target-skill rehearsal and explicit generalisation support—did not independently explain variation in functional effect size. At the same time, the stable self-regulation effect across construct coverage levels should be read alongside contemporary measurement evidence showing that self-regulation instruments differ substantially in their coverage of the cognitive, behavioural and emotional regulation domains and their constituent components [22]. The resulting challenge for the field is therefore not merely whether self-regulation can be changed, but how well-defined regulatory gains become a transferable, usable function in children’s everyday lives.

## 5. Conclusions

This systematic review and meta-analysis provide evidence that non-pharmacological self-regulation-focused interventions can improve both self-regulation and separate child functional outcomes in autistic children and young people. Effects on self-regulation were consistently positive, while functional effects were more variable and strongly graded by their proximity to the intervention target. Functional outcomes that were directly targeted or practised showed substantially larger effects than distal outcomes requiring transfer beyond the immediate intervention content; however, the positive distal effect indicates that benefits can extend beyond what is explicitly trained. Comparator design contributed to variation in observed effects, whereas target-skill rehearsal and explicit generalisation support did not independently explain functional effect-size differences. These findings highlight the need for future trials to distinguish proximal regulatory change, target-aligned function and distal functional generalisation and to use outcome measures that clearly specify the self-regulation constructs and functional domains being assessed.

## Figures and Tables

**Table 1:** Characteristics of included studies.

Study Author, Year, Design, Country; Risk-of-Bias Rating (RoB-2)	Aim	Eligibility Criteria (Diagnosis Method; Diagnostic Tool; IQ; IQ Tool; Inclusion Criteria)	Group (n = Sample Size); Characteristics (Mean Age + *SD*; Male%)	Outcome Alignment (Domains of Self-Regulation or Function)	Respondent/Evaluation Method: Outcome Measure	Results	
						Immediate Post-Intervention	Follow-Up
Afsharnejad et al., 2022 [70]: RCT; Australia Some concerns of bias	Efficacy of KONTAKT^®^ in supporting various meaningful social skills goals.	*Diagnosis:* DSM-IV [83] or DSM-5 [53] diagnosis of ASD; *Tool (ASD):* ADOS-II [84] *IQ:* >70; *Tool (IQ):* WASI-II [85] *Inclusion:* 12 to 17 years, located in Perth, nil co-occurring psychiatric conditions.	***(I)* Intervention** KONTAKT^®^ (n = 46); *Age:* 13.9 + 1.7; *Male%:* 72. ***(II)* Control** Super Chef (n = 44); *Age:* 13.7 + 1.5; *Male%:* 73.	Domain-level (CR, ER) Component-level measure (CR domain)	*Self:* Emotion Regulation Social Skills Questionnaire (ERSSQ) [86] *Direct task:* Mindreading Battery [87]	No significant difference between groups identified on *p* values or effect sizes (*ES*) on ERSSQ (*p* = .62, *ES* = 0.11) and Mindreading Battery emotion recognition accuracy (*p* = .71, *ES* = 0.08) measures.	At 12-week follow-up, significant within-group improvements on ERSSQ (*p* = .03, *ES* = 0.20) and Mindreading Battery (*p* = .001, *ES* = 0.31) for both groups.
				Social function (getting along) Quality of life (mobility, getting along, life activities)	*Self:* Goal Attainment Scaling *Parent and self:* Paediatric Quality of Life Inventory 4.0™ (PedsQL 4.0™) [88]	No significant difference between groups identified; however, intervention and control groups made significant progress (*p* < .001) towards their GAS goals. No significant between-group or within-group effects identified on PedsQL 4.0™.	Significant treatment effect identified on GAS at follow-up, *p* = .04, *ES* = 0.35. No significant between-group or within-group effects identified on PedsQL 4.0™ at follow-up.
Beaumont & Sofronoff, 2008 [86]: RCT; Australia High risk of bias	Evaluate a programme aiming to enhance emotional understanding and social skills.	*Diagnosis:* DSM-IV-TR [89] diagnosis of Asperger syndrome by paediatrician; *Tool (ASD):* CAST [90] *IQ:* ≥85; *Tool (IQ):* WISC-III Short-form [91]; *Inclusion:* Aged 7.5 to 11 years.	***(I)* Intervention** Junior Detective Training Programme (n = 26); *Age:* 9.64 + 1.21; *Male%:* 88.46. ***(II)* Control** Waitlist (n = 23); *Age:* 9.81 + 1.26; *Male%:* 90.30.	Domain-level (CR, ER) Component-level measure (CR domain)	*Parent:* Emotion Regulation Social Skills Questionnaire (ERSSQ) [86] *Direct task:* Assessment of Emotion Perception from Facial Expression [92] and Posture Cues [93]; Dylan is Being Teased [94]; James and the Maths Test [95]	Mixed-model MANOVA identified a significant overall main effect of group, *F*(2, 45) = 6.52, *p* < .01, η^2^ = 0.23 and significant group by time interaction revealed on parent-reported social skill measures (ERSSQ and SSQ-P). Mixed-model MANOVA identified a significant main effect of time on both emotion perception measures, *F*(2, 45) = 10.13, *p* < .001, η^2^ = 0.31 and significant main effect of group, *F*(2, 45) = 6.82, *p* < .01, η^2^ = 0.23, and time, *F*(2, 45) = 11.57, *p* < .001, η^2^ = 0.34 and a group by time interaction on the knowledge of emotion management strategies (Dylan and James measures).	Significant improvement from pre-to 6-week and 5-month follow-up for intervention and control groups on ERSSQ. Direct task scores continued to improve for all intervention and control groups across direct tasks; however, improvements were not statistically significant.
				Social function (getting along)	*Parent:* Social Skills Questionnaire (SSQ-P) [96]	Refer to ERSSQ results.	Significant improvement on SSQ-P identified at 6-week and 5-month follow-up for intervention and control groups.
Beaumont et al., 2021 [97]: RCT; Australia Some concerns of bias	Effect of a parent-supported social skills programme.	*Diagnosis:* DSM-5 [53] formal ASD diagnosis by a paediatrician, psychiatrist or clinical psychologist; *Tool (ASD):* NR; *IQ:* Average range; *Tool (IQ):* NR; *Inclusion:* 7 to 12 years, living in QLD, NSW, VIC. Individuals with ADHD or ODD required appropriate management.	***(I)* Intervention** Secret Agent Society (n = 35); *Age:* 9.80 + 1.57; *Male%:* 86.0. ***(II)* Control** Central Intelligence Agency (CIA) (n = 35); *Age:* 9.99 + 1.16; *Male%:* 87.0.	Domain-level (CR, ER)	*Parent and teacher:* Emotion Regulation Social Skills Questionnaire (ERSSQ-P; ERSSQ-T) [86]	Significant main effect for time and main effect for group on ERSSQ-P and ERSSQ-T. A significant group by time interaction was identified on ERSSQ-P only. Mean improvement was significantly greater for intervention group than the control condition on ERSSQ-P, *t*(1, 68) = 4.20, *p* < .001, partial η^2^ = 0.20.	At 6-week follow-up, a significant improvement in mean ERSSQ-P scores remained at 6-week follow-up for ERSSQ-P only, *t*(1, 68) = 3.46, *p* < .001, partial η^2^ = 0.15.
				Social function (getting along)	*Parent and teacher:* Social Skills Questionnaire (SSQ-P; SSQ-T) [96]	Significant main effect for time, main effect for group and a group by time interaction on SSQ-P and SSQ-T. Mean improvement was significantly greater for the intervention group on SSQ-P, *t*(1, 68) = 4.40, *p* < .001, η_p_^2^ = 0.22.	Differences in the mean improvement of parent-reported SSQ between groups remained significant at follow-up, *t*(1, 68) = 3.47, *p* < .001, η_p_^2^ = 0.15. SSQ-T not evaluated at follow-up.
Cahart et al., 2022 [60]: RCT; United Kingdom Some concerns of bias	Impact of drum training on behaviour and brain function.	*Diagnosis:* Clinical DSM-5 [53] diagnosis of ASD; *Tool (ASD):* AQ [98]; *IQ:* Within normal range; *Tool (IQ):* WASI-II [85]; *Inclusion:* Aged 16 to 19 years, right-handed, nil learning disabilities.	***(I)* Intervention** Drum Training (n = 19); *Age:* 17.59 + 1.29; *Male%:* 64.7. ***(II)* Control** Treatment as usual (n = 17); *Age:* 17.93 + 1.48; *Male%:* 73.3.	Component-level measure (BR and CR domains)	*Parent:* Social Skills Improvement System—Rating Scales (SSIS) [99] Hyper-activity/Inattention subscale	Significant group by time interaction on SSIS Hyperactivity/Inattention subscale, *F*(1, 34) = 9.56, *p* = .004, η^2^ = 0.22.	NA
				Social function (getting along)	*Parent:* Social Skills Improvement System—Rating Scales (SSIS) [99] Overall Social Skills score	Not reported in main report.	NA
Clifford et al., 2022 [100]: RCT; Netherlands Some concerns of bias	Effect of an attention-based cognitive behavioural treatment on anger coping and aggressive behaviour.	*Diagnosis:* Primary diagnosis of ASD by experienced clinicians; *Tool (ASD):* ADOS [101]; *IQ:* In normal range; *Tool (IQ):* NR; *Inclusion:* Aged 8 to 13 years, individuals with ADHD required no changes to medication.	***(I)* Intervention** Anger Can Go! (n = 27); *Age:* 10.2 + 1.58; *Male%:* 76.9. ***(II)* Control** Waitlist (n = 24); *Age:* 10.2 + 1.56; *Male%:* 75.0.	Domain-level (CR, ER) Component-level measure (ER domain)	*Parent and self:* Behaviour Anger Response Questionnaire (BARQ-P; BARQ-C) [102] *Parent and teacher:* Questionnaire Social Behaviour (QSB) [100]	Statistically significant treatment effect on BARQ-P adaptive anger coping strategies, *R*^2 =^ 0.17, *p* = .001; however, no treatment effect identified on BARQ-C, *R*^2 =^ 0.03, *p* = .151. No significant treatment effects on parent- (*R*^2 =^ 0.113, *p* = .036) or teacher- (*R^2^* ^=^ 0.010, *p* = .364) reported QSB.	NA
				Quality of life (mobility, getting along, life activities)	*Self:* Paediatric Quality of Life 4.0 Inventory™ (PedsQL 4.0™) [88]	No treatment effect was found on Peds-QL 4.0™, *p* = .845, *R*^2^ = 0.001.	NA
de Vries et al., 2015 [73]: RCT; Netherlands High risk of bias	Effect of working memory and flexibility training interventions compared to an active control.	*Diagnosis:* Prior DSM-IV [83] diagnosis of ASD by a multidisciplinary team; *Tool (ASD):* SRS [103,104], ADI-R [105]; *IQ:* ≥80; *Tool (IQ):* WISC-III [89]; *Inclusion:* Aged 8 to 12 years, nil co-occurring seizure conditions.	***(I)* Intervention** Working Memory Training (n = 40); *Age:* 10.5 + 1.4; *Male%:* 87.1. ***(II)* Intervention** Cognitive Flexibility Training (n = 40); *Age:* 10.7 + 1.4; *Male%:* 92.6. ***(III)* Control** Mock-training (n = 40); *Age:* 10.5 + 1.4; *Male%:* 93.8.	Global/multi- domain (BR, CR, ER) Component-level measure (CR domain)	*Parent:* Behaviour Rating Inventory of Executive Function^®^ (BRIEF^®^) [106] *Direct task:* Corsi block tapping task (Corsi-BTT) [107]; Gender-emotion switch task [108]; N-back task [109,110]; Number-gnome switch task [111]; Stop-task [112]; Sustained attention response task (SART) [113]	Significant effect for time identified on BRIEF^®^ total, *F*(2, 168) = 17.9, *p* < .001, η_p_^2^ = 0.18. A significant effect of time on Corsi-BTT (*p* < .001), SART (*p* < .001) and some reaction time and error rate values for the N-back task and Gender-emotion switch task (*ps* < .01) were observed. Significant differences (*ps* < .01) in pre- and post-intervention scores were identified for all direct task measures with the exception of the Number-gnome switch task and Stop-task. No significant time by intervention interactions found with the exception of the Corsi-BTT measure (*p* < .05).	At 6-week follow-up, significant differences between pre- and post-treatment values were not maintained (*ps* > .01). Differences in pre-training and follow-up values were significant at follow-up for the N-back task measure only. No significant differences identified for the remaining direct tasks.
				Quality of life (mobility, getting along, life activities)	*Parent:* Paediatric Quality of Life Inventory 4.0™ (PedsQL 4.0™) [88]	Significant effect of time identified on PedsQL 4.0™, *F*(2, 168) = 8.6, *p* < .001, η_p_^2^ = 0.09. Across groups, significant difference between pre- and post-intervention scores was found (*ps* < .01).	Significant differences were not maintained at follow-up.
Factor et al., 2019 [61]: RCT; United States of America Some concerns of bias	Effect of STAMP on child lability and negativity, ER and parental confidence managing child’s negative affect.	*Diagnosis:* Meets ASD criteria by clinical judgement with corroborating diagnostic test scores; *Tool (ASD):* ADOS [101] or ADOS-II [84]; *IQ:* NR; *Tool (IQ):* KBIT [114]; *Inclusion:* Aged 4 to 7 years, parent-reported difficulties with managing anger or anxiety.	***(I)* Intervention** Stress and Anger Management Programme (n = 12); *Age:* 5.54 + 0.94; *Male%:* 75.0. ***(II)* Control** Waitlist (n = 11); *Age:* 5.36 + 1.12; *Male%:* 90.9.	(I) Domain-level (CR, ER)	*Parent:* Emotion Regulation Checklist (ERC) [115]	Significant decrease in ERC lability/negativity (ERC-L/N) subscale scores for treatment group, *t*(9) = 2.45, *p* = .03, no significant difference in scores for waitlist group. No significant differences in ERC emotion regulation (ERC-ER) subscale scores for treatment, *t*(9) = 0.00, *p* = 1.0, or waitlist groups, *t*(7) = 0.72, *p* = .49.	At 9-week follow-up, the difference in scores from pre-to follow-up did not reach significance on ERC-ER or ERC-L/N subscales.
				Social function and prosocial behaviour (getting along)	*Parent:* Strengths and Difficulties Questionnaire (SDQ) [116]	Within both groups, a significant change in total internalising and externalising behaviours (SDQ) from pre-to post-treatment, *t*(5) = 3.07, *p* = .03, was identified.	NR
Fatta et al., 2024 [62]: RCT; Italy Some concerns of bias	Efficacy of PEERS^®^ on social abilities, co-occurring conditions and executive functions.	*Diagnosis:* Certified ASD Level 1 diagnosis confirmed by clinical staff; *Tool (ASD):* ADOS-II [84]; *IQ:* ≥70; *Tool (IQ):* WISC-IV [117]; *Inclusion:* Aged 12 to 18 years, Italian language fluency, reported social difficulties, nil co-occurring medical or psychiatric conditions.	***(I)* Intervention** PEERS^®^ (n = 18); *Age:* 15.14 + 2.26; *Male%:* 66.7. ***(II)* Control** Waitlist (n = 19); *Age:* 15.50 + 1.74; *Male%:* 73.7.	Global/multi- domain (BR, CR, ER)	*Parent:* Behaviour Rating Inventory of Executive Function, second edition (BRIEF^®^2) [118]	No treatment effect reported. Significant group by time interaction effect, with large effect size identified on BRIEF^®^2 ER Index, *F*(1, 22) = 15.20, *p* < .001, η_p_^2^ = 0.41, for treatment group only. No significant interaction effects for CR or BR indexes. Significant difference in pre- and post-treatment BRIEF^®^2 ER Index values reported, *F*(1, 36) = 16.53, *p* < .001, η_p_^2^ = 0.31 with large effect size.	At 3 months follow-up, a significant difference with large effect between pre- and follow-up scores was identified on the BRIEF^®^2 Global Executive Composite, *F*(1, 36) = 12.05, *p* < .001, η_p_^2^ = 0.25. Differences remained significant for ER Index, *F*(1, 36) = 18.63, *p* < .001, η_p_^2^ = 0.34.
				Social performance (getting along)	*Parent and self:* Quality of Socialisation Questionnaire-Revised (QSQ-R) [119,120]	Significant increase in QSQ-R Social Initiative scale only for treatment group in parent- (*z* = −3.412, *p* < .001) and adolescent-report (*z* = −3.086, *p* = .002) questionnaires. No statistically significant differences identified on QSQ-R Social Reciprocity scale for intervention or control groups.	Significant differences in parent- and adolescent-reported QSQ-R social initiative scale scores maintained at follow-up. Additionally, a statistically significant increase in adolescent-reported QSQ-Social Reciprocity score was reported for the treatment group.
Gabriels et al., 2015 [121]: RCT; United States of America Some concerns of bias	Effect of THR on self- regulation, communication, social, adaptive and motor behaviours.	*Diagnosis:* Confirmed by meeting clinical cut-offs for ASD; *Tools (ASD):* ADOS [101] or ADOS-II [84]; *IQ:* ≥40; *Tool (IQ):* Leiter-R-Brief [122]; *Inclusion:* Aged 6 to 16 years, meet Aberrant Behaviour Checklist-Community [123] threshold score, nil medical or behaviour concerns.	***(I)* Intervention** Therapeutic Horseback Riding (n = 63); *Age:* 10.5 + 3.2; *Male%:* 84.5. ***(II)* Control** Barn Activity (n = 64); *Age:* 10.0 + 2.7; *Male%:* 89.7.	Component-level measure (ER domain)	*Parent:* Aberrant Behaviour Checklist-Community (ABC-C) [123]	Statistically significant between-group differences to medium effect size on Irritability (*p* = .02, *d* = 0.50) and Hyperactivity (*p* = .01, *d* = 0.53) subscales (ABC-C). No significant between-group differences on remaining ABC-C subscales.	NA
				Adaptive function (cognition, mobility, self-care, getting along, life activities, participation)	*Parent:* Vineland Adaptive Behavioural Scales, second edition (VABS-2) [124]	No statistically significant between-group differences were identified for the Adaptive total score (VABS-2), (*p* = .64, *d* = 0.11).	NA
Kenworthy et al., 2014 [65]: Cluster RCT; United States of America Some concerns of bias	Effect of an executive function intervention in comparison to a social skills intervention.	*Diagnosis:* Meet the ADOS [99] diagnostic ASD threshold and meet DSM-IV-TR [87] criteria for PDD; *Tool (ASD):* ADOS [101]; *IQ:* >70; *Tool (IQ):* WASI [125]; *Inclusion:* Aged 7 to 12 years, verbal mental age > 8 years.	***(I)* Intervention** Unstuck and on Target (n = 47; n = 10 schools); *Age:* 9.49 + 1.00; *Male%:* 87. ***(II)* Control** Social skills programme (n = 20; n = 4 schools); *Age:* 9.58 + 1.10; *Male%:* 90.	Global/multi- domain (BR, CR, ER) Component-level measure (CR domain)	*Parent and teacher:* Behaviour Rating Inventory of Executive Function^®^ (BRIEF^®^) [106] *Direct task:* Challenge Task (CT) [65]; WASI [125] Block Design subtest	Statistically significant treatment effects were identified on parent-and teacher-reported BRIEF^®^ Shift, (*d* = −0.66, 95% CI [0.15, 1.24]; *d* = −0.89, CI [−1.62, −0.33]), and Plan/Organise, (*d* = −0.45, CI [−0.97, 0.07]; *d* = −0.57, CI [−1.26, 0.01]), subscales respectively. Treatment effects were also reported for CT flexibility subscale, *d* = −0.72, CI [−1.38, 0.14], and WASI Block Design, *d* = 0.65, CI [0.18, 1.17], scores.	NA
				School function (life activities)	*Blinded observer:* Classroom observations [65]	Improvement in classroom behaviours reported for both groups, with stronger improvements in the treatment group participants.	NA
Kirst et al., 2022 [126]: RCT; Germany High risk of bias	Evaluate the impact of a parent-assisted serious game on emotion recognition and empathy.	*Diagnosis:* ICD-10 [54] diagnosis of ASD, Asperger’s syndrome or PDD-NOS by a multi-disciplinary team; *Tool (ASD):* ADI-R [105] and SCQ [127]; *IQ:* >70; *Tool (IQ):* Coloured Progressive Matrices [128]; *Inclusion:* Aged 5 to 10 years, German language verbal age < 5.0, nil co-occurring medical conditions or aggressive behaviour.	***(I)* Intervention** Zirkus Empathico (n = 42); *Age:* 8.1 + 1.6; *Male%:* 76.2. ***(II)* Control** Parent-assisted computerised training (n = 40); *Age:* 7.6 + 1.3; *Male%:* 92.5.	Domain-level (CR, ER) Component-level measure (CR domain) Component-level measure (ER domain)	*Parent:* Emotion Regulation Checklist (ERC) [115] *Direct task:* Kids Emotion Recognition Multiple Images Task (KERMIT) [129] *Self:* Level of Emotional Awareness Scale for Children (LEAS-C) [130]	Intervention effect was reported for ERC-L/N (*DI* = −3.3, *d* = −0.47) and ERC-ER (*DI* = 2.3, *d* = 0.70) subscales. Between-group difference in KERMIT accuracy scores favouring the intervention group (*DI* = 2.0, *d* = 0.50) with medium effect size. Intervention effect identified on LEAS-C (*DI* = 0.3, *d* = 0.67) scores.	At 3-month follow-up, an intervention effect was maintained on ERC-ER (*DI* = 1.2, *d* = 0.33) and LEAS-C measure (*DI* = 0.1, *d* = 0.24) for the treatment group, but not on the ERC-L/N (*DI* = 0.2, *d* = 0.03) subscale. The statistically significant between-group difference on KERMIT was not maintained (*DI* = −0.7, *d* = −0.18).
				Quality of life/well-being (mobility, getting along, life activities)	*Parent:* Kiddy Kindl [131]	Non-significant positive effects reported on Kiddy Kindl for the treatment group, *DI* = 2.0, *d* = 0.18.	Positive effects remained non-significant at follow-up, *DI* = 1.5, *d* = 0.14.
Lopata et al., 2010 [132]: RCT; United States of America High risk of bias	Efficacy of a comprehensive social intervention on social and behaviour ratings.	*Diagnosis:* Documented ASD, PDD-NOS or Asperger’s syndrome diagnosis, confirmed by two senior researchers; *Tool (ASD):* NR; *IQ:* >70; *Tool (IQ):* WISC-IV Short form [117]; *Inclusion:* Aged 7 to 12 years, meet expressive and receptive language threshold, nil physical aggression.	***(I)* Intervention** Manualised Social Intervention (n = 18); *Age:* 9.39 + 1.72; *Male%:* 94.4. ***(II)* Control** Waitlist (n = 18); *Age:* 9.56 + 1.54; *Male%:* 94.4.	Global/multi- domain (BR, CR, ER) Component-level measure (CR domain)	*Parent and clinician:* Adapted Skillstreaming Checklist (ASC) [133] *Direct task:* Diagnostic Analysis of Nonverbal Accuracy 2 (DANVA-2) [134]	Medium to large treatment effects identified for parent-, *p* = .006, *d* = 0.0584, and staff-report ASC, *p* < .001, *d* = 1.421. Treatment effects were reported as non-significant; however, a medium effect size favouring the treatment group was reported on DANVA-2, *d* = 0.532, *p* = .024.	NA
				Social function (getting along)	*Parent and staff:* Behaviour Assessment System for Children, second edition—Parent and Teacher Rating Scales (BASC-2-PRS; BASC-2-TRS) [135]	Medium to large treatment effect identified for Social Skills subscale on BASC-2-TRS, *p* = .001, *d* = 0.776 and Withdrawal subscale on BASC-2-PRS, *p* < .001, *d* = 1.055, and BASC-2-TRS, *p* = .007, *d* = 0.693. Non-significant positive effects on BASC-2-PRS Social Skills subscale, *p* = .044, *d* = 0.365.	NA
Lopata et al., 2016 [136]: RCT; United States of America Low to some concerns of bias	Efficacy of a computer-based intervention as an additional component of a psychosocial treatment.	*Diagnosis:* Clinical diagnosis of ASD, Asperger’s syndrome or PDD-NOS; *Tool (ASD):* ADI-R [105]; *IQ:* >70; *Tool (IQ):* WISC-IV Short form [117]; *Inclusion:* Aged 7 to 12 years, absence of significant behaviour problems.	***(I)* Intervention** summerMAX with Mind Reading (n = 18); *Age:* 8.83 + 1.47; *Male%:* 94.4. ***(II)* Control** summerMAX (n = 18); *Age:* 8.83 + 1.50; *Male%:* 94.4.	Global/multi- domain (BR, CR, ER) Component-level measure (CR domain)	*Parent and clinician:* Adapted Skillstreaming Checklist (ASC) [133] *Parent and clinician:* Emotion recognition and display survey (ERDS) [137]; *Direct task:* Cambridge mind reading face-voice battery for children (CAM-C) [138]	Significant main effects for time were found on ASC parent-, *p* < .001, ω^2^ = 0.585, and clinician-ratings, *p* < .001, ω^2^ = 0.468, with large effect. Statistically significant main effects for time were found for the CAM-C faces *p* < .001, ω^2^ = 0.298, and voices *p* = .002, ω^2^ = 0.199, scales and ERDS parent-, *p* < .001, ω^2^ = 0.394, and clinician-ratings, *p* < .001, ω^2^ = 0.369, with large effect.	NA
				Social function (getting along)	*Parent and clinician:* Behaviour Assessment System for Children, second edition—Parent and Teacher Rating Scales (BASC-2-PRS; BASC-2-TRS) [132]	Significant main effects for time were found on the BASC-2-TRS Social Skills subscale, *p* < .001, ω^2^ = 0.298, with large effect size. Additionally, medium effect was reported for the BASC-2-PRS Social Skills subscale, *p* = .012, ω^2^ = 0.117.	NA
Lopata et al., 2019 [66]: Cluster RCT; United States of America Low to some concerns of bias	Efficacy of a manualized comprehensive school-based intervention.	*Diagnosis:* Diagnosis of ASD; *Tool (ASD):* ADI-R [105]; *IQ:* >70; *Tool (IQ):* WISC-IV Short form [117]; *Inclusion:* Aged 6 to 12 years, nil intellectual and language disability.	***(I)* Intervention** schoolMAX (n = 52; n = 17 schools); *Age:* 8.65 + 1.29; *Male%:* 90.4. ***(II)* Control** Treatment as usual (n = 51; n = 18 schools); *Age:* 9.01 + 1.45; *Male%:* 92.2.	Global/multi- domain (BR, CR, ER) Component-level measure (CR domain)	*Parent and teacher:* Adapted Skillstreaming Checklist (ASC) [133] *Direct task:* Cambridge mind reading face-voice battery for children (CAM-C) [138]	Statistically significant between-group differences for ASC parent-teacher ratings, *F*(1, 100) = 32.91, *p* = .001, *d* = 1.29, with large effect. Significant treatment effect identified on CAM-C, *F*(1, 100) = 33.16, *p* < .001, *d* = 1.41, with large effect.	NA
				Social function (getting along)	*Blinded observer:* Social Interaction Observation Scale (SIOS) [139]	No statistically significant between-group differences were reported for SIOS, *F*(1, 100) = 0.16, *p* = .962, *d* = 0.08.	NA
Lopata et al., 2021 [71]: RCT; United States of America Low to some concerns of bias	Efficacy of a comprehensive outpatient psychosocial treatment with autistic children.	*Diagnosis:* Diagnosis of ASD confirmed using ADI-R [103]; *Tool (ASD):* ADI-R [105]; *IQ:* >70; *Tool (IQ):* WISC-IV Short-form [117]; *Inclusion:* Aged 7 to 12 years, meet expressive and receptive language threshold.	***(I)* Intervention** MAXout (n = 44); *Age:* 9.77 + 1.76; *Male%:* 86. ***(II)* Control** Waitlist (n = 44); *Age:* 9.57 + 1.73; *Male%:* 84.	Global/multi- domain (BR, CR, ER) Component-level measure (CR domain)	*Parent:* Adapted Skillstreaming Checklist (ASC) [133] *Direct task:* Cambridge mind reading face-voice battery for children (CAM-C) [138]	Significant treatment effects identified on ASC immediately post-intervention, *p* < .001, *d* = 0.74, 95% CI [0.39, 1.09], with medium effect size. Group by time effect was not statistically significant for CAM-C.	At 4–6 weeks follow-up, treatment effects were maintained, *p* = .001, *d* = 0.59, with medium effect size. Group by time effect remained non-significant.
				Social function (getting along)	*Parent:* Behaviour Assessment System for Children, third edition—Parent Rating Scale (BASC-3-PRS) [140]; *Blinded observer:* Social Interaction Observation Scale (SIOS) [139]	Small treatment effects were identified on BASC-3-PRS Social Skills subscale, *d* = 0.39, 95% CI [0.06, 0.72], and Behaviour Symptoms Index (BSI), *d* = 0.40, 95% CI [0.09, 0.71]. Group by time effect was not statistically significant for SIOS.	Treatment effects identified on BASC-3-PRS Social Skills, *d* = 0.38, CI [0.05, 0.72], and Behaviour Symptoms Index, *d* = 0.35, CI [0.04, 0.66], were maintained at follow-up. Group by time effect remained non-significant.
Marzouki et al., 2022 [74]: RCT; North Africa Some concerns of bias	Effect of two aquatic training programmes on locomotion, stereotypy skills and emotion regulation.	*Diagnosis:* Certified ASD diagnosis meeting DSM-5 [53] criteria and CARS [140] score >30; *Tool (ASD):* CARS [141]; *IQ:* ≥80; *Tool (IQ):* Wechsler Intelligence Scale for Adolescents [117]; Verbal subtests; *Inclusion:* Aged 6 to 7 years, confirmed diagnosis of ASD, nil physical or developmental impairments, severe behavioural problems or prescribed medications interfering with learning or motor performance.	***(I)* Intervention** Technical Aquatic Activities (n = 10); *Age:* 6.3 + 0.5; *Male%:* 87.5. ***(II)* Intervention** Game-Based aquatic activities (n = 10); *Age:* 6.4 + 0.5; *Male%:* 100.0. ***(III)* Control** Treatment as usual (n = 8); *Age:* 6.3 + 0.5; *Male%:* 83.3.	Domain-level (CR, ER)	*Parent:* Emotion Regulation Checklist (ERC) [115]	Group by time interaction effect was not statistically significant for either ERC subscale. Main effect for time identified for ERC-L/N subscale, *F*(1, 19) = 10.120, *p* < .005, *ES* = 0.659, for treatment groups and ERC-ER subscale for all intervention and control conditions, *F*(1, 19) = 26.272, *p* < .0001, *ES* = 1.097.	NA
				Motor activity performance (mobility)	*Direct task:* Test of Gross Motor Development 2 (TGMD-2) [142]	A significant group and time interaction effect was identified across both intervention groups for TMGD-2 Locomotor Skills, *F*(1, 19) = 25.995, *p* < .0001, *ES* = 1.507, and Control Skills subscales, *F*(2, 19) = 44.360, *p* < .001, *ES* = 1.985. Significant main effect for time reported for both intervention groups compared with the control for both TMGD-2 subscales.	NA
Morgan et al., 2018 [67]: Cluster RCT; United States of America Some concerns of bias	Efficacy of classroom-based Social Communication, Emotional Regulation, and Transactional Supports (SCERTS) intervention.	*Diagnosis:* Clinical or educational DSM-IV [83] diagnosis of ASD, Asperger’s syndrome or PDD-NOS; *Tool (ASD):* ADOS [101]; *IQ:* NR; *Tool (IQ):* Stanford-Binet Intelligence Scale (5th Ed) [143]; *Inclusion:* Enrolled in kindergarten to grade 2 in a general or special education setting, nil motor delay/impairment, dual sensory impairment and no history of traumatic brain injury.	***(I)* Intervention** Classroom SCERTS (n = 118; n = 34 schools); *Age:* 6.82 + 1.07; *Male%:* 78.80. ***(II)* Control** Autism Training Modules (n = 79; n = 26 schools) *Age:* 6.77 + 1.02; *Male%:* 84.80.	Global/multi- domain (BR, CR, ER)	*Teacher:* Behaviour Rating Inventory of Executive Function^®^ (BRIEF^®^) [106]	Significant between-group differences on BRIEF^®^ Global Executive Composite score, *p* < .001, *d* = −0.40.	NA
				Adaptive function (mobility, self-care, getting along, life activities) Classroom engagement (getting along, life activities) Social function (getting along)	*Parent:* Vineland Adaptive Behavioural Scales—second edition (VABS-2) [124] *Blinded observer:* Classroom Measure of Active Engagement (CMAE) [144] *Teacher:* Social Skills Rating System (SSRS) [145]	Significant between-group differences on one VABS-2 subscale (communication), *p* < .05, *d* = 0.31. Small to moderate treatment effect identified on CMAE Social Interaction composite, *F*(1, 49.57) = 6.24, *p* < .05, *d* = 0.34. No treatment effect identified on the CMAE Instructional Participation composite. Significant between group differences on social skills subscale (SSRS) favouring the intervention, *p* < .01, *d* = −0.45. Significant between group differences on the SSRS Problem Behaviour subscale favouring the active control, *p* < .01, *d* = −0.36.	NA
Soorya et al., 2015 [146]: RCT; United States of America Low to some concerns of bias	Efficacy of a targeted social skills intervention on social cognition and social behaviour.	*Diagnosis:* ASD diagnosis meeting DSM-IV [83] criteria; *Tool (ASD):* ADOS [101], ADI-R [105]; *IQ:* >70; *Tool (IQ):* NR; *Inclusion:* 8 to 11 years, nil new psychiatric medications, nil active seizure disorders or aggression toward others.	***(I)* Intervention** Seaver-NETT (n = 35); *Age:* 10.05 + 1.27; *Male%:* 85.7. ***(II)* Control** Facilitated play (n = 34); *Age:* 9.87 + 1.32; *Male%:* 84.38.	Component-level measure (CR domain)	*Direct task:* Diagnostic Analysis of Nonverbal Accuracy-2 (DANVA-2) [147]; Reading the Mind in the Eyes Test (RMET) [148]	No significant time and group interaction effect identified on the social cognition composite, which includes DANVA-2, RMET and Strange Stories Task [149], *B* = 0.00 (0.13), *p* = .98.	At 3-month follow-up, no significant interaction effect was found on the social cognition composite, *B* = −0.17 (0.17), *p* = .31.
				Social function (getting along)	*Parent:* Children’s Communication Checklist-2 (CCC-2) [150]	Significant time and group interaction effect reported, for the social behaviour composite (Griffith Empathy Measure [151] and CCC-2), for the intervention group (*d* = 0.88) compared to the control group (*d* = 0.12), *B* = −0.31 (0.14), *p* = .04.	No significant interaction effect on the social behaviour composite, *B* = −0.14 (0.19), *p* = .47, at follow-up.
Thomeer et al., 2012 [152]: RCT; United States of America Some concerns of bias	Efficacy of a comprehensive psychosocial intervention with autistic children.	*Diagnosis:* Prior clinical diagnosis of ASD, Asperger’s syndrome or PDD-NOS; *Tool (ASD):* ADI-R [105]; *IQ:* >70; *Tool (IQ):* WISC-IV Short form [117]; *Inclusion:* Aged 7 to 12 years, meet expressive and receptive language threshold, nil physical aggression.	***(I)* Intervention** summerMAX (n = 17); *Age:* 9.24 + 1.64; *Male%:* 82.4. ***(II)* Control** Waitlist (n = 18); *Age:* 9.39 + 1.91; *Male%:* 88.9.	Global/multi- domain (BR, CR, ER) Component-level measure (CR domain)	*Parent and clinician:* Adapted Skillstreaming Checklist (ASC) [133] *Direct task:* Diagnostic Analysis of Nonverbal Accuracy-2 (DANVA-2) [134]	Large treatment effects were identified on parent-report ASC, *p* = .001, *d* = 0.85, and clinician-rated, *p* < .001, *d* = 1.57, ASC. Non-significant results on DANVA-2 (*p* = .078, *d* = 0.34) favouring the intervention condition.	At 2–3 months follow-up, a small treatment effect was identified on the parent-rated ASC, *p* = .006, *d* = 0.47. DANVA-2 not evaluated at follow-up.
				Social function (getting along)	*Parent and teacher:* Behaviour Assessment System for Children, second edition—Parent and Teacher Rating Scales (BASC-2-PRS; BASC-2-TRS) [134]	Treatment effects were found on BASC-2-PRS and BASC-2-TRS: Withdrawal (*p* = .020, small effect *d* = 0.39; *p* = .012, medium effect *d* = 0.78) and Social Skills scales (*p* = .011, medium effect *d* = 0.70; *p* = .003, large effect *d* = 0.84) respectively.	Medium treatment effect on BASC-2-PRS Social Skills scale found at follow-up, *p* = .004, *d* = 0.68, Withdrawal scale results were non-significant. BASC-2-TRS not evaluated at follow-up.
Thomeer et al., 2015 [153]: RCT; United States of America Some concerns of bias	Efficacy of a computer software and in vivo rehearsal intervention.	*Diagnosis:* Prior clinical diagnosis of ASD, Asperger’s syndrome or PDD-NOS; *Tool (ASD):* ADI-R [105]; *IQ:* >70; *Tool (IQ):* WISC-IV Short form [117]; *Inclusion:* Aged 7 to 12 years, meet expressive and receptive language threshold, nil physical aggression.	***(I)* Intervention** Mind Reading (n = 22); *Age:* 8.86 + 1.39; *Male%:* 86.4. ***(II)* Control** Waitlist (n = 22); *Age:* 8.57 + 1.16; *Male%:* 90.5.	Component-level measure (CR domain)	*Direct task:* Cambridge Mindreading Face-Voice Battery for Children (CAM-C) [138]; *Parent:* Emotion recognition and display survey (ERDS) [137]	Moderate to large treatment effects on CAM-C Faces (*p* < .001, ω^2^ = 0.23) and Voices subscales *(p* < .001, ω^2^ = 0.14), and the ERDS Expressive (*p* = .0025, ω^2^ = 0.11) and Receptive (*p* = .006, ω^2^ = 0.08) subscales.	At 5-week follow-up, significant between-group differences were maintained (*ps* = .05), on both CAM-C subscales and the ERDS Expressive subscale. For the ERDS Receptive subscale, a significant between-groups difference was reported at follow-up, *p* = .038, *d* = 0.73.
				Social function (getting along)	*Parent:* Behaviour Assessment System for Children, second edition—Parent Rating Scale (BASC-2-PRS) [135]	No significant between-group differences identified on BASC-2-PRS Social Skills scale, *p* = .168, ω^2^ = 0.00.	BASC-2-PRS not evaluated at follow-up.
Thomeer et al., 2019 [154]: RCT; United States of America Some concerns of bias	Feasibility and effect of a comprehensive psychosocial treatment when implemented by a community agency.	*Diagnosis:* Prior clinical diagnosis of ASD, Asperger’s syndrome or PDD-NOS; *Tool (ASD):* SCQ [127]; *IQ:* >70; *Tool (IQ):* WISC-IV Short form [117]; *Inclusion:* Aged 7 to 12 years, meet expressive and receptive language threshold.	***(I)* Intervention** summerMAX (n = 28); *Age:* 8.74 + 1.44; *Male%:* 89.3. ***(II)* Control** Waitlist (n = 29); *Age:* 9.55 + 1.77; *Male%:* 79.3.	Global/multi- domain (BR, CR, ER)	*Parent and clinician:* Adapted Skillstreaming Checklist (ASC) [133]	Significant treatments effect identified for parent-, *p* < .001, medium effect *d* = 0.75 and clinician-rated ASC, *p* < .001, large effect *d* = 1.75.	NA
				Social function (getting along)	*Parent and teacher:* Behaviour Assessment System for Children, second edition—Parent and Teacher Rating Scales (BASC-2-PRS; BASC-2-TRS) [135]	Treatment effects found on BASC-2-PRS and BASC-2-TRS Social Skills scale, (*p* = .002, small effect *d* = 0.46; *p* < .001, large effect *d* = 1.28), and BASC-2-PRS Withdrawal scale, (*p* = .003, small effect *d* = 0.31; *p* = .007, medium effect *d* = −0.54), respectively.	NA
Wan Yunus et al., 2021 [63]: RCT; Malaysia Some concerns of bias	Examine the effect of self-regulated learning on challenging behaviour and school-related function.	*Diagnosis:* Formal DSM-5 [53] diagnosis of ASD by paediatrician; *Tool (ASD):* NR; *IQ:* ≥50; *Tool (IQ):* NR; *Inclusion:* Aged 6 to 12 years, a raw score of > 10 on Vinelands-II [124] Maladaptive Behaviour Index, attending school or home school.	***(I)* Intervention** Self-regulated Learning (n = 20); *Age:* 7.70 + 1.49; *Male%:* 75. ***(II)* Control** Activity-based control (n = 20); *Age:* 7.45 + 1.19; *Male%:* 95.	Global/multi- domain (BR, CR, ER)	*Parent:* Behaviour Rating Inventory of Executive Function^®^ (BRIEF^®^) [106]	Large treatment effects identified on both BRIEF^®^ BR (*p* = .00, η^2^ = 0.73) and Metacognition (*p* = .00, η^2^ = 0.72) indexes.	At 1-month follow-up, large treatment effects were maintained on both BRIEF^®^ BR (*p* = .001, η^2^ = 0.72) and Metacognition indexes (*p* < .001, η^2^ = 0.74).
				Adaptive function (mobility, self-care, getting along, life activities) School function (life activities)	*Parent and teacher:* Vineland Adaptive Behaviour Scale (Maladaptive Behaviour index), second edition (VABS-2) [124] *Parent and teacher:* School Function Assessment (SFA) [155]	Large treatment effect identified on VABS-2 Maladaptive Behaviour index (*p* = .002, η^2^ = 0.68). Large treatment effects (η^2^ > 0.138) were found across SFA all domains (*ps* < .001) including the Personal Care Awareness domain (*p* = .03).	Large treatment effects were maintained at follow-up on VABS-2 Maladaptive Behaviour index (*p* = .001, η^2^ = 0.71). All large treatment effects were sustained at follow-up.
Wang et al., 2025 [156]: RCT; China High risk of bias	Effect of fundamental movement skills intervention practice on executive function and social interaction.	*Diagnosis:* Clinical diagnosis of moderate ASD by hospital institutions; *Tool (ASD):* NR; *IQ:* NR; *Tool (IQ):* NR; *Inclusion:* Child enrolled in first-grade, nil physical conditions, not engaged in other exercise activities.	***(I)* Intervention** Fundamental Movement Skills (n = 13); *Age:* 7.75 + 1.65; *Male%:* 92.3. ***(II)* Control** Treatment as usual (n = 12); *Age:* 7.63 + 1.58; *Male%:* 91.7.	Global/multi- domain (BR, CR, ER)	*Parent and teacher:* Behaviour Rating Inventory of Executive Function^®^—second edition (BRIEF^®^2) [118]	Statistically significant between-group difference on BRIEF^®^2 total scores (*p* < .001, large effect *d* = −2.773). In the BR Index, all sub-dimensions (Inhibition, Shifting, Emotional Control) reached statistical significance (*ps* < .01) with large effect size. Among the Metacognition Index subdomains, only the Working Memory sub-dimension revealed a statistically significant between-group difference (*p* = .001), with large effect size.	NA
				Motor activity performance (mobility)	*Direct task:* Test of Gross Motor Development 3 (TGMD-3) [157]	Significant improvement in TGMD-3 Locomotor Skills subscale identified for the treatment group, *d* = 0.951, *p* < .05. Improvement on TGMD-3 Manipulation subscale was not statistically significant (*p* > .05).	NA
Weiss et al., 2018 [72]: RCT; Canada High risk of bias	Efficacy of a manualised, cognitive behaviour therapy intervention on emotional regulation and mental health.	*Diagnosis:* Documented ASD diagnosis from a qualified clinician; *Tool (ASD):* SCQ-Lifetime Version [127], SRS-2 [158], ADOS [101]; *IQ:* ≥79; *Tool (IQ):* WASI-II [85]; *Inclusion:* 8 to 12 years, parent-reported difficulties managing emotions.	***(I)* Intervention** Secret Agent Society: Operation Regulation (n = 35); *Age:* 9.63 + 1.26; *Male%:* 88.6. ***(II)* Control** Waitlist (n = 33); *Age:* 9.88 + 1.29; *Male%:* 87.9.	Domain-level (CR, ER) Component-level measure (CR domain)	*Parent:* Emotion Regulation Social Skills Questionnaire (ERSSQ) [86]; Emotion Regulation Checklist (ERC) [115]; *Self:* Children’s Emotion Management Scales (CEM) [159] *Direct task:* Dylan is Being Teased [94]; James and the Maths Test [95]	Significant treatment effect on ERC-L/N subscale, *p* = .04, medium effect *d* = 0.58, and on the ERSSQ, *p* < .01, large effect *d* = 0.79. No significant between-group differences for ERC-ER subscale or the CEM measure. No significant between-group differences reported for James and the Maths Test (*p* = .11, *d* = 0.38) or Dylan is Being Teased (*p* = .62, *d* = 0.12) measures.	At 10-week follow-up, treatment gains were maintained across ERC-L/N, ERSSQ measures. No significant between-group effects identified on any direct task measures at follow-up.
				Social function (getting along)	*Parent:* Behaviour Assessment System for Children, second edition—Parent Rating Scales (BASC-2-PRS) [135]	Significant effects of intervention were identified on the BASC-2-PRS Adaptive composite, *p* < .04, medium effect *d* = 0.71, and Behavioural Symptoms Index, *p* = .04, medium effect *d* = 0.52.	Treatment gains were maintained across the BASC-2-PRS Adaptive and Behavioural Symptom composites only.
Williams et al., 2012 [160]: RCT; Australia Low to some concerns of bias	Effect of an emotion training programme on emotion recognition skills, theory of mind and social skills.	*Diagnosis:* DSM-IV [81] clinical diagnosis of ASD by a paediatrician; *Tool (ASD):* ADOS [101]; *IQ:* equivalent to or above 30 months of age; *Tool (IQ):* WPPSI-III [161]; *Inclusion:* Aged 4 to 7 years, not previously watched The Transporters programme.	***(I)* Intervention** The Transporters (n = 28); *Age (months):* 62.83 + 11.17; *Male%:* 89.3. ***(II)* Control** Thomas the Tank Engine DVD (n = 27); *Age (months):* 61.93 + 9.91; *Male%:* 85.2.	Component-level measure (CR domain)	*Direct task:* Emotion identification and emotion matching tasks [162]; Developmental Neuropsychological Assessment, second edition (NEPSY-II) [163] Affect Recognition and Theory of Mind tasks; Situational and Desire-based Mindreading tasks [164]	Significant effect of intervention by time identified for the emotion identification, *B* = 0.73 (0.26), *p* = .01, and emotion matching, *B* = 1.68 (0.59), *p* < .0011. No significant intervention by time effects for the NEPSY-II or mind-reading tasks.	At 3-month follow-up, significant intervention by time interaction (post-intervention to follow-up) for the happiness emotion identification task, *B* = 0.60 (0.26), *p* = .02, and on the Mindreading Situational task, *B* = 1.10 (0.51), *p* = .03. Post-treatment improvements in anger emotion identification and NEPSY-II Theory of Mind task were not maintained at follow-up; both tasks demonstrated a significant decrease in scores, *B* = −0.68 (0.29), *p* = .02 and *B* = −0.99 (0.38), *p* = .01, respectively.
				Social function (getting along)	Vineland Adaptive Behavioural Scales—second edition (VABS-2) [124] Socialisation domain	No significant intervention by time interaction on VABS-2 Socialisation scores.	NR

Note. ADHD = attention-deficit/hyperactivity disorder; ADI-R = Autism Diagnostic Interview-Revised; ADOS = Autism Diagnostic Observation Schedule; ADOS-II = Autism Diagnostic Observation Schedule, Second Edition; AQ = Autism Quotient; ASD = autism spectrum disorder; CARS = Childhood Autism Rating Scale; CAST = Childhood Asperger Spectrum Test; DSM-IV = Diagnostic and Statistical Manual of Mental Disorders, Fourth Edition; DSM-IV-TR = Diagnostic and Statistical Manual of Mental Disorders, Fourth Edition, Text Revision; DSM-5 = Diagnostic and Statistical Manual of Mental Disorders, Fifth Edition; ICD-10 = International Classification of Diseases, Tenth Revision; IQ = intelligence quotient; KBIT = Kaufman Brief Intelligence Test; Leiter-R-Brief = Leiter International Performance Scale-Revised, Brief; NR = not reported; NSW = New South Wales; ODD = oppositional defiant disorder; PDD = pervasive developmental disorder; PDD-NOS = pervasive developmental disorder not otherwise specified; QLD = Queensland; SCQ = Social Communication Questionnaire; SRS = Social Responsiveness Scale; SRS-2 = Social Responsiveness Scale, Second Edition; VIC = Victoria; WASI = Wechsler Abbreviated Scale of Intelligence; WASI-II = Wechsler Abbreviated Scale of Intelligence, Second Edition; WISC-III = Wechsler Intelligence Scale for Children, Third Edition; WISC-IV = Wechsler Intelligence Scale for Children, Fourth Edition; WPPSI-III = Wechsler Preschool and Primary Scale of Intelligence, Third Edition. Italics and bold are used within this table for emphasis, to distinguish between study characteristics and aid readability purposes.

**Table 2:** Summary of self-regulation-based interventions for autistic children and young people.

Intervention; References	Self-Regulation Domain/s (BR, CR, ER)	Aim	Interventionist	Dosage/Mode of Delivery (Clinician:Child Ratio)/Setting	Tailoring/Modifications	Content, Procedures and Materials
**KONTAKT^®^** Afsharnejad et al., 2022 [70]	BR, CR	Targeting self-confidence, communication, social motivation and interaction skills, and problem-solving skills.	Therapist (at least one clinician was a certified KONTAKT^®^ trainer). *Qualifications*: Occupational therapy, psychology, education, social work backgrounds. *Training*: 3-day training course and supervision and feedback.	Dosage: 24 h. *Child sessions:* 1 × 90 min session per week for 16 weeks over 2 school terms. **Mode:** Child group (2:6–8) **Setting:** Clinic	Manuals and workbooks were translated from Swedish to English. Adapted to a 16-session variant from 24-sessions. Goal setting, and intervention delivery frameworks were also developed to ensure delivery consistency. Intervention content (i.e., role-play scenarios, games, discussion topics) were modified to suit the Australian cultural context. Homework “missions” were also individualised to each participant’s goals.	KONTAKT^®^ [165,166] is a manualized programme that utilised elements of cognitive behaviour therapy (CBT), parent involvement, behavioural activation and observational learning. *Child:* Sessions followed a structured framework. Session opening, review of group rules and homework, group discussions and activities, snack time, assigning new homework and closedown.
**Junior Detective Training Programme (JDTP)** Beaumont et al., 2008 [86]	BR, CR, ER	Targets social competence including emotion recognition, emotional regulation abilities as well as social interaction.	Therapist *Qualifications*: Psychology or counselling backgrounds. *Training*: NR	**Dosage:** *Child:* 13 h. *Computer game:* 1 × 45–60 min session a week for 4 weeks. *Group session:* 1 × 60–120 min session a week for 7 weeks. *Parent:* 9.5 h. *Group sessions:* 1 × 60–120 min session a week for 7 weeks. **Mode:** Combined child and parent group Child group Parent group **Setting:** Clinic	Sessions involved varied amounts of the computer game and small group components as the intervention progressed. JDTP: 60 min during session 1 and 2. 45 min during session 3 and 4. No JDTP in sessions 4 to 7. Group sessions and parent sessions: 60 min during session 1, 2 and 7. A total of 75 min during session 3 and 4. A total of 120 min during session 5 and 6.	JDTP is a computer game that involves the participant acts as a junior detective to decode “suspects” thoughts and feelings. The intervention supports children with developing emotion identification skills, relaxation and mindfulness technique knowledge, problem-solving skills, play skills and ways to handle bullying, cope with mistakes, manage uncertainty and try new things, manage frustration. *Child intervention components:* 1. JDTP (combined group): The computer game involved progressing through 3 levels that increased in difficulty. 2. Group sessions (children only): targeted generalisation of the computer game content and teach problem-solving and social skills. 3. Home missions: involving identifying own emotions and practicing strategies to regulate emotions. 4. Secret Agent Journal: reward system implemented to reinforce mission completion and desired behaviour. *Parent:* group sessions were delivered concurrently with child groups and aimed to support parent understanding of the programme content and homework and teach strategies. *Materials:* handouts for teachers, posters, visual support cards.
**Secret Agent Society (SAS) Adaptation** Beaumont et al., 2021 [97]	CR, ER	Targets social and emotional skills and generalisation of learned skills.	Parent *Qualifications*: NR. *Training*: Parents attended a 150 min webinar, received programme delivery guide and attended weekly video-chat support group sessions (up to 3 parents).	**Dosage:** total hours not reported. *Child sessions*: 1 session a week for 10 weeks. **Mode:** Child individual **Setting:** Home	Parents facilitated sessions instead of trained therapists. A programme delivery guide was provided to parents to facilitate the weekly activities. Adaptations to the SAS [167] program included reducing program materials and small group content was adapted.	The adapted SAS programme was parent facilitated. Sessions targeted emotion recognition, theory of mind, relaxation strategies, social skills, and managing bullying. *Child intervention components:* 1. SAS: refer to “JDTP” intervention component. 2. Activities involving the SAS visual support cards 3. Home missions: encouraged skill practice and generalisation on a daily basis. *Materials:* parent and teacher handouts, visual support cards.
**Drum Training** Cahart et al. 2022 [60]	*BR, CR*	Hyperactivity, attention, problem behaviours and repetitive and restricted behaviours.	Professional drum tutor *Qualifications*: Drum tutor had knowledge of the Rockschool syllabus. *Training*: NR	**Dosage:** 12 h *Child sessions:* 2 × 45 min sessions per week for 8 weeks. **Mode:** Child individual **Setting:** Participant’s home or music studio.	The pace of content delivery was individualised to each participant and dependent on their drumming abilities and progress.	*Child:* Drum tutors followed the standardised Rockschool drumming syllabus for beginners during intervention sessions.
**Anger Can Go!** Clifford et al., 2022 [100]	BR, CR, ER	Aims to improve emotional (anger) regulation in autistic children through developing attention control, attention shifting, emotional awareness, awareness of expressed emotion and knowledge of coping strategies.	Therapist *Qualifications*: psychologists. *Training*: received training delivered by senior clinical psychologists and supervision sessions.	**Dosage:** *Child:* 9 h. *Child sessions:* 1 × 60 min session a week for 9 weeks. *Parent:* total hours not reported. *Parent sessions:* 3 sessions delivered. *Teacher:* dosage not reported. **Mode:** Child individual Parent group Teacher individual **Setting:** Clinic	**Anger Can Go! Parent Only Adaptation** An adaptation of Anger Can Go! [168] was delivered in this study. In this variation, only the three parent psychoeducation sessions were delivered.	Anger Can Go! is a dialectical behaviour therapy (DBT) and mindfulness cognitive behaviour therapy (MCBT) approach. Intervention involves implementing a “time-out” procedure for the child to engage in learned coping strategies. *Child:* sessions focus on pictures and stories involving a role model character named Bob. Children are also provided a workbook. In the final 10 min of the session, the child provides a session outline to their parent and are assigned homework. *Parents:* provided psychoeducation to increase awareness of expressed emotion before child sessions. *Teachers:* school visit completed to introduce time-out procedure.
**Braingame Brian** de Vries et al., 2015 [73]	BR, CR	Executive functions specifically, working memory (WM), flexibility, inhibition and their transfer to daily life.	Parent (informal facilitator) *Qualifications*: NR. *Training*: Parents received information about Braingame Brian and training instructions prior to intervention.	**Dosage:** 18.75 h. *Child sessions:* A total of 5 × 45 min sessions per week (maximum one per day) for 6 weeks. A total of 25 sessions. **Mode:** Child individual **Setting:** Online, virtual world via laptop	**WM training adaptation:** WM tasks were adaptive to performance and success. While flexibility and inhibition tasks were not trained (remained at low level). **Flexibility training adaptation:** cognitive flexibility tasks were adaptive to performance and success. While WM and inhibition tasks were not trained.	Braingame Brian is a digital-based training game where the child is an inventor in a virtual world. *Child:* Within a 45 min training session, two training blocks last 15 min. Each training block involved 3 tasks on WM, cognitive flexibility and inhibition.
**Stress and Anger Management Programme (STAMP)** Factor et al., 2019 [61]	CR, ER	Support emotional regulation development including strategies to manage emotions and reduce distress.	Clinician *Qualifications*: clinical graduate students and staff members. *Training*: received training and supervision by a licenced clinical psychologist.	**Dosage:** *Child:* 9 h. *Child sessions:* 1 × 60-min session a week for 9 weeks. *Parent:* 9 h. *Parent sessions:* 1 × 60 min session a week for 9 weeks. **Mode:** Child group (1:2–3) Parent group **Setting:** Clinic	STAMP is an adaptation of the Exploring Feelings [169] CBT programme. Child sessions had a shorter duration. Intervention content was adjusted to include songs, stories and play activities. Parent training was added.	STAMP [170] intervention incorporated education on affect, various tools (i.e., physical, relaxation, social, thinking, special interest) understanding of emotion expression. *Child:* sessions involved welcome time, singing, story time, activity/lesson time, snack and goodbyes. Each session would target a different topic (i.e., affect and emotion education or emotional regulation/problem solving tools). *Homework tasks:* Provided weekly for parent and child to complete. *Parent:* Caregiver sessions included psychoeducation, review of intervention content and homework tasks. Parents also met with a therapist outside of clinic. *Materials:* Parent handout with overview of intervention sessions.
**Programme for the Education and Enrichment of Relational Skills PEERS^®^** [171] Fatta et al., 2024 [62]	CR	Targets social skills including the ability to make and maintain friendships, manage conflict.	Clinician *Qualifications*: clinical psychology, psychotherapy, clinical psychology trainee backgrounds. *Training*: attended 3-day training with certified provider.	**Dosage:** *Child: 28* h. *Child sessions:* 1 × 2 h session a week for 14 weeks. *Parent: 28* h. *Parent sessions:* 1 × 2 h session a week for 14 weeks. **Mode:** Child group Parent group **Setting:** Telehealth	PEERS^®^ [171] intervention materials were translated to Italian and adapted to the Italian cultural context. Role-playing videos used during sessions were transcribed and adapted and the original videos were subtitled. Handouts were provided to adolescents. Session materials and activities were modified for telehealth delivery. Homework tasks were completed during sessions.	The PEERS^®^ Italian Adaptation is a social skills training intervention that targets social skill development. Adolescent and parent telehealth sessions occurred concurrently. *Child:* each of the fourteen sessions had a different focus. Sessions involved psychoeducation, reviewing role-playing videos, cognitive strategies, behavioural rehearsal exercises and performance feedback. *Parent:* trained parents on their ability to provide social coaching with their child in natural settings. *Materials:* Parent and adolescent handouts and homework sheets.
**Therapeutic Horseback Riding (THR)** Gabriels et al., 2015 [121]	BR	Improve and maintain self-regulation, social, communication, motor and adaptive behaviours.	Therapeutic riding instructor *Qualifications*: PATH International [172] advanced therapeutic riding instructor certification. *Training*: NR.	**Dosage:** 7.5 h. *Child sessions:* 1 × 45 min session a week for 10 weeks. **Mode:** Child group (3:2) **Setting:** Therapeutic riding facility	None reported.	The manualised equine-assisted activities utilised behavioural teaching methods. *Child:* Sessions followed a regular routine and focused on (1) therapeutic riding skills (e.g., horse mounting, halting, steering, turning and trotting) and (2) horsemanship skills (e.g., how to lead and care for a horse).
**Unstuck and on Target (UOT)** [173] Kenworthy et al., 2014 [65]	BR, CR, ER	Improve executive functioning, physical/mental flexibility, goal setting, planning and related behaviours.	School staff *Qualifications*: NR *Training*: Initial two-hour course and 6 monthly trainings.	**Dosage:** *Child:* total hours not reported. *Child sessions:* 28 × 30–40 min sessions over a year. *Parent:* total hours not reported. *Parent sessions:* 2 sessions. *Teacher:* total hours not reported. *Teacher sessions:* 1 session. **Mode:** Child group (1–2:3–6) Parent group Teacher group **Setting:** School	None reported.	The CBT-based intervention aimed to teach participants about various cognitive skills (i.e., physical/mental flexibility, goal setting and planning). *Child:* Sessions involved demonstrations, visuals, videos and discussions. Skills taught during sessions were also applied in role plays, games and activities. *Parent and classroom teachers:* Addressed the programme, targeted skills and autism phenomenology. Parents additionally received a manual outlining how to model and support flexibility and planning scripts at home. *Materials:* Visual cues.
**Zirkus Empathico** Kirst et al., 2022 [126]	CR, ER	Foster development of empathic behaviour and emotion understanding in real-world settings.	Caregiver *Qualifications*: NR. *Training*: caregivers received a training manual, on-site instruction and weekly supervision.	**Dosage:** total hours not reported. *Child sessions*: minimum 100 min (minimum) a week for 6 weeks. **Mode:** Child individual **Setting:** Online via tablet at home	Modules are accessed according to the child’s progress.	The manualised tablet-based serious game addressed self-awareness of emotions, emotion recognition, inference and understanding appropriate reactions toward other people’s emotions. Caregivers discussed content and supported skill generalisation. *Child intervention components:* *Serious game:* Modules 1 to 4 incorporated video stimuli and emotion eliciting situations videos within the game. *Module 5:* 10 min per day (at a minimum) targeted skill transfer of skills targeted in Modules 1 to 4. *Paper-based reward system:* used for reinforcement motivation.
**summerMAX** Lopata et al., 2010 [132] Lopata et al., 2016 [136] Thomeer et al., 2012 [152] Thomeer et al., 2019 [154]	BR CR	Teach emotion recognition, broader emotional and social functioning including social skills, social communication, interest expansion, emotion recognition as well as address ASD-associated symptoms.	Therapist *Qualifications*: psychology and education graduate and undergraduate students. *Training*: All staff clinicians received intensive training and demonstrated 90% fidelity.	**Dosage:** *Child:* 145.83 h. *Child sessions:* 25 × 70 min treatment cycles per week (5 sessions per day) for 5 weeks. *Weekly outing:* not reported. *Parent:* 7.5 h. *Parent training:* 1 × 90 min session a week for 5 weeks. **Mode:** Child group (3:6) Parent group **Setting:** Clinic	**Lopata et al., 2010** [132]. **Adaptations (Manualised Social Intervention)** Children were grouped by age: 7–8 years, 9–10 years, 11–12 years. Skills targeted were adjusted depending on age group. **Lopata et al., 2016** [136]. **Adaptations (summerMAX + MR)** *Group-Based Intervention*: 3 × 70 min treatment cycles per week, children received the Mind Reading [152] treatment protocol. (See “Mind Reading”). Response-cost system: reinforcers included computer time in addition to edible snacks. **Thomeer et al., 2012** [152] **Adaptations** *Response-cost system:* no tangible rewards provided as positive reinforcement. *Individualised Daily Note (IDN):* IDN: three to four goals addressed. **Thomeer et al., 2019** [154] **Adaptations** **Mode:** Child group (5–6 children). **Setting:** community clinic. *Group-Based Intervention*: During treatment cycles, 25 min was allocated to skill instruction, and the remaining 45 min to therapeutic activity.	The CBT-based manualised intervention involved: *Child intervention components:* 1. Group-based intervention: Treatment cycles targeted social-communication, social skills, non-literal language skills and interest expansion. Treatment cycles consisted of: (a) Intensive skill instruction (20 min) followed the *Skillstreaming* [174,175] 9-step instructional sequence. (b) Therapeutic activity (50 min) to practice and reinforce the development of the targeted skill. Activities required at least two children to work together to solve a problem/successfully complete the activity. 2. Response-cost system: provided reinforcement (positive and negative) according to engagement. Encouraged use of newly learned skills and adherence to group rules. Every 20 min, a therapist provided point-based feedback to each child in a group-based format. 3. Individualised Daily Note (IDN): Targeted two to three individualised goals. At the end of a treatment day, if IDN targets were achieved, children received a reinforcer (e.g., edible snack) in the clinic and at home (e.g., preferred activity). 4. Weekly outing: a weekly outing allowed for skill practice in real-world settings. Outings were earned using weekly response-cost points and IDN goal performance. *Parent:* training provided psychoeducation, manualised programme information, and behaviour reinforcement and skill generalisation strategies.
**schoolMAX** Lopata et al., 2019 [66]	BR, CR	Targets social skills, communication, emotion recognition, nonliteral language skills, and interest expansion.	School staff *Qualifications*: psychologist, speech pathologist, social worker, classroom aide, teaching assistant, counsellor, teacher background. *Training*: received training to learn about the intervention prior to delivering the intervention and met weekly for the duration of the intervention.	**Dosage:** *Child*: total hours not reported. *Social skills group:* 2–3 × 60–90 min sessions per week. *Mind Reading:* 2–3 sessions totalling 60 min duration per week for a year. *Therapeutic activities:* 2 × 20–30 min sessions per week for a year. *Parent:* total duration not reported. *Parent sessions:* 1 × 60–90 min session a month, for 12 months. **Mode:** Child group Parent group **Setting:** School	An adapted version of the summerMAX [132] intervention for delivery in the school setting. Response-cost system was removed. Group-based intervention was delivered as two separate groups (i.e., social skills sessions and therapeutic activities). Mind Reading [153] intervention was added.	The CBT-based manualised intervention targeted skills including emotion recognition, interpersonal and prosocial skills. *Child intervention components:* 1. Social Skills Sessions: followed the *Skillstreaming* [174,175] syllabus and aimed to teach interpersonal skills. 2. Emotion Recognition Instruction (Mind Reading [153]): Monitored by a teaching assistant. Children engaged in Mind Reading which aimed to teach emotion recognition through facial and vocal expressions of 10 groups of 10 emotions. See “Mind Reading” 3. Therapeutic Activities: Involved practice of social and emotion recognition skills and expand interests. 4. Individual Daily Note (IDN): implemented all school days. The behavioural reinforcement system targeted three goals to encourage targeted skill generalisation. *Parent:* Monthly sessions included the following content: programme information, teaching strategies to use across various settings.
**MAXout** Lopata et al., 2021 [71]	BR, CR	Targets social skills, communication, emotion recognition, nonliteral language skills, and interest expansion.	Therapist *Qualifications*: *Training*: provided and fidelity was monitored throughout treatment implementation.	**Dosage:** *Child:* 54 h. *Child sessions:* 2 × 90 min sessions (2 × 45 min treatment cycles per session) a week for 18 weeks. *Parent:* 6 h. *Parent sessions:* 1 × 90 min session every 3 weeks, for 18 weeks. **Mode:** Child group (2:4) Parent group **Setting:** Clinic	An adapted version of the summerMAX [132] intervention for delivery as an intensive outpatient treatment. Treatment cycle duration was reduced to-45 min (15 min intensive instruction and 30 min therapeutic activity) Response-cost system: feedback was provided every 15 min.	See “summerMAX” intervention.
**Technical Aquatic Activities** Marzouki et al., 2022 [74]	ER	Locomotion, stereotypy and emotional regulation.	Qualified swimming trainer *Qualifications*: experience with adapted physical education and certified to work with autistic children. *Training*: NR.	**Dosage:** 13.3 h *Child sessions:* 2 × 50 min sessions a week for 8 weeks. **Mode:** Child group (1:2) **Setting:** Local indoor swimming pool	None reported.	The training protocol was based on the four phases of the Halliwick Method [176] and targeted foundational swimming skills including “flapping kicks, forward crawling strokes and sideways head turns” [72]. *Child*: sessions involved a 5 min (out of pool) warm-up, 7 min pool warm-up, 30 min programme and 8 min cool-down. *Materials*: gross motor equipment.
**Game-based Aquatic Activities** Marzouki et al., 2022 [74]	ER	Locomotion, stereotypy and emotional regulation.	See “Technical Aquatic Activities”	See “Technical Aquatic Activities”	Modifications made to Pan’s [177] intervention protocol: shorter session duration, 1:1 instruction removed.	Based on the training protocol in Pan’s study [177]. The intervention targeted social interaction, motor skill development, communication, cooperation and water confidence. *Child:* see “Technical Aquatic Activities”. Warm-up involved game-based cooperative activities.
**Social Communication, Emotional Regulation, and Transactional Supports (SCERTS)** Morgan et al., 2018 [67]	BR, CR, ER	Targets active engagement in social interaction and learning activities in the classroom.	Teacher *Qualifications*: NR. *Training*: Teachers attended a 3-day (18 h total) intensive training course and received regular coaching twice monthly (at a minimum). Fidelity checklist utilised.	**Dosage:** *Child:* 25 h per week for 8 months across a school year. **Mode:** Child group **Setting:** School	None reported.	The naturalistic developmental behavioural intervention (NDBI)focuses on social communication, ER, and transactional support (i.e., learning support for the child and providing support to families and between professionals). The intervention involved embedding principles into activities and was used in conjunction with the existing school curriculum. The SCERTS model consists of three elements: (1) SCERTS assessment process, (2) Classroom SCERTS intervention (CSI), and (3) coaching for teacher facilitators.
**Seaver-NETT** Soorya et al., 2015 [146]	BR, CR	Targets social cognitive impairment experienced by autistic children, including non-verbal communication, emotion recognition, theory of mind, social skills and thought management.	Therapist *Qualifications*: therapist had clinical psychologist background. *Training*: Therapists received a training, supervision and a manual.	**Dosage:** *Child:* 18 h. *Child sessions:* 1 × 90 min session a week for 12 weeks. *Parent:* 6 h. *Parent sessions:* 1 × 30 min session a week for 12 weeks. **Mode:** Child group (1:2) Parent group **Setting:** Clinic	None reported.	The manualised intervention followed a social skills curriculum drawing from various approaches. The intervention targeted three skills: non-verbal communication, emotion recognition and theory of mind. Instructional strategies include behaviour reinforcement, role-play, skill practice and group activities. *Child:* sessions followed a specific structure that included play/snack time, group instruction and wrap-up/circle time. *Parent:* involved learning about the programme, reviewing homework and discussing barriers or challenges faced during group sessions.
**Mind Reading (MR)** Thomeer et al., 2015 [153]	BR, CR	Teach recognition of simple and complex emotions.	Therapist *Qualifications*: psychology or education background. *Training*: Facilitators received eight hours of training and supervision.	**Dosage:** 36 h. *Child sessions:* 2 × 90 min sessions per week for 12 weeks. **Mode:** Child group **Setting:** Clinic (computer lab)	None reported.	The manualised intervention programme involves three elements aiming to teach the recognition of 412 emotions. Each session consists of five 15-to-20 min intervals of activities. *Child intervention components:* 1. Mind Reading: interactive software to learn the recognition of emotions. 2. In vivo rehearsal trials: to practice emotion recognition and promote skill maintenance and generalisation. 3. Behaviour reinforcement system: to appropriately reinforce behaviour during sessions.
**Self-Regulated Learning (SRL) Intervention** Wan Yunus et al., 2021 [63]	BR, CR, ER	Self-monitoring, including organisation, prioritisation, sustaining focus, regulating alertness, managing emotions, working memory and recall and monitoring and self-regulating action.	Therapist *Qualifications*: paediatric occupational therapy background. *Training*: NR.	**Dosage:** 12 h. *Child sessions*: 1 × 1 h session a week for 12 weeks. **Mode:** Child individual **Setting:** Clinic	None reported.	Self-regulated learning intervention targeted task performance across 15 daily and school tasks. *Child:* sessions involved following a 9-step procedure involving review and task analysis of a pre-recorded video of an adult completing a task. The child would then be videotaped completing the same task before completing task analysis of their own performance and identifying any solutions to task errors.
**Fundamental Movement Skills Practice (FMS)** Wang et al., 2025 [156]	BR, CR	Improving social communication and executive function.	Teacher *Qualifications:* NR. *Training*: NR.	**Dosage:** 54 weeks. *Child sessions:* 4 × 45 min sessions per week for 18 weeks. **Mode:** Child group (2:5) **Setting:** Special education school	None reported.	Intervention was guided by a model of sequential motor skill progression. Intervention targeted 21 fundamental movement skills including stability, locomotor and manipulation skills. *Child:* sessions involved a 5 min warm-up, 35 min practicing basic motor skills and 5 min for equipment cleaning and dismissal. *Materials*: gross motor equipment.
**Secret Agent** **Society: Operation Regulation (SAS:OR)** Weiss et al., 2018 [72]	CR, ER	Targeted emotion regulation abilities including emotion recognition and awareness, coping strategy awareness in autistic children.	Clinician *Qualifications*: clinical psychology graduate student or postdoctoral fellow. *Training*: Intervention facilitators attended a one-day workshop and weekly supervision.	**Dosage:** 10 h. *Combined sessions:* 1 × 60 min session per week for 10 weeks. **Mode:** Child and parent combined session **Setting:** Clinic	Removed the social skills curriculum and incorporated additional activities to improve emotional regulation.	The transdiagnostic CBT-based intervention adapted the SAS [167] intervention. SAS:OR [178] utilised the same theme as the original intervention. *Combined sessions:* involved select computer games from SAS programme, emotion education activities, and code cards. Parents were encouraged to engage in sessions. *Token system:* to maintain motivation and attention. *Home missions:* weekly planned exposures to distressing situations in home and school settings. *Home-school diary:* to support skill maintenance and generalisation of targeted behaviours. *Materials*: child and parent workbooks, teacher handouts and a home-school diary.
**The** **Transporters** Williams et al., 2012 [160]	CR	Teach emotion recognition skills to children with autism aged 3 to 8 years.	Parent (informal facilitator) *Qualifications*: NR. *Training*: Parents received parent user guides, to support their child in completing the quizzes.	**Dosage:** 1.25 h. *Child sessions:* 7 × 15 min sessions a week for 4 weeks. **Mode:** Child individual **Setting:** Home	None reported.	*Child:* The Transporters consisted of fifteen 5 min episodes designed to teach recognition of 15 emotions. Each episode had an interactive quiz to reinforce learning.

Note. ASD = autism spectrum disorder; BR = behaviour regulation; CR = cognitive regulation; ER = emotion regulation; NR = not reported. Italics and bold are used within this table for emphasis, to distinguish between study characteristics and aid readability purposes.

**Table 3:** Principal meta-analysis and key moderator results.

Outcome/Analysis	*k*	Estimate	95% CI	*τ*	*Q*_β_ and *p*
**SELF-REGULATION (primary meta-analysis: 27 intervention-comparator effects; 25 independent studies)**					
**Overall pooled effect**	**27**	***g* = 0.605**	**0.454 to 0.756**	**0.270**	**—**
***Moderator: Comparator type***					
Active/attention comparator	13	*g* = 0.444	0.297 to 0.590	0.108	*Q*_β_*=* 12.234, *p* = .002
Treatment as usual	5	*g* = 0.747	0.021 to 1.474	0.685	
Waitlist/no treatment	9	*g* = 0.878	0.682 to 1.074	0.000	
***Moderator: Self-regulation construct coverage***					
Component-level	6	*g* = 0.553	0.337 to 0.768	0.036	*Q*_β_ = 0.883, *p* = .643
Domain-level	9	*g* = 0.719	0.438 to 1.000	0.282	
Global/multi-domain	12	*g* = 0.583	0.317 to 0.849	0.357	
**FUNCTIONAL OUTCOMES**					
**Overall pooled effect—primary synthesis**	**25**	***g* = 0.527**	**0.356 to 0.698**	**0.320**	**—**
***Independent-study functional moderator dataset (23 independent studies)***					
***Moderator: Comparator type***					
Active/attention comparator	11	*g* = 0.335	0.145 to 0.525	0.216	*Q*_β_ = 7.167, *p* = .028
Treatment as usual	4	*g* = 0.727	−0.021 to 1.476	0.660	
Waitlist/no treatment	8	*g* = 0.739	0.502 to 0.977	0.179	
***Primary functional moderator: Outcome proximity***					
Direct/target-aligned	13	*g* = 0.761	0.496 to 1.027	0.382	*Q*_β_ = 11.034, *p* < .001
Distal/generalisation	10	*g* = 0.250	0.108 to 0.393	0.000	
***Mechanism moderator: Supported application + generalisation***					
No	10	*g* = 0.409	0.138 to 0.681	0.324	*Q*_β_ = 0.918, *p* = .338
Yes	13	*g* = 0.578	0.365 to 0.792	0.290	
***Mechanism moderator: Target-skill application/rehearsal***					
No	8	*g* = 0.354	0.031 to 0.678	0.345	*Q*_β_ = 1.353, *p* = .245
Yes	15	*g* = 0.578	0.385 to 0.770	0.278	
***Mechanism moderator: Explicit generalisation support***					
No	9	*g* = 0.430	0.128 to 0.731	0.352	*Q*_β_ = 0.622, *p* = .430
Yes	13	*g* = 0.578	0.365 to 0.792	0.290	
***Sensitivity analysis: Outcome proximity restricted to active/attention comparators***					
Direct/target-aligned	4	*g* = 0.659	0.220 to 1.099	0.367	*Q*_β_ = 3.770, *p* = .052
Distal/generalisation	7	*g* = 0.196	0.038 to 0.355	0.000	

Note. Italics and bold are used within this table for emphasis, to distinguish between study characteristics and aid readability purposes. k denotes the number of intervention–comparator comparisons for the principal pooled analyses and self-regulation moderator analyses. For functional moderator analyses, k denotes the number of independent study-level effects after combining the de Vries et al. [73] and Marzouki et al. [74] multi-arm intervention groups, yielding 23 independent studies. The explicit generalisation-support analysis included 22 studies because one unclear classification was treated as missing. *Q*_β_ denotes the mixed-effects between-subgroup Q test. The 95% prediction intervals for the principal pooled effects were 0.027 to 1.183 for self-regulation and −0.158 to 1.212 for functional outcomes. Full subgroup prediction intervals, τ^2^ estimates, subgroup *Z* tests, and sensitivity-analysis results are reported in the Supplementary Material.

## Data Availability

The data analysed in this review were extracted from published studies. The study-level data and coding underlying the meta-analyses are provided in the Supplementary Materials.

## Supplementary Materials

The following supporting information can be downloaded at https://www.mdpi.com/article/10.3390/children13101415/s1, Table S1: Search strategies by database; Table S2: Principal pooled analyses: full statistical reporting; Table S3: Sensitivity analyses; Table S4: Categorical moderator analyses—full statistical reporting; Table S5: Meta-regression results; Table S6: Small-study-effect diagnostics for self-regulation and functional outcomes; Table S7: Functional mechanism coding definitions; Table S8: Study-level inputs for multi-arm functional moderator analyses; Table S9: Final study-level functional moderator coding; Table S10: Final inferential status of functional mechanism variables; Table S11: Self-regulation construct alignment; Table S12: Protocol-to-final-review summary; Table S13: Outcome-selection decisions for studies with multiple eligible outcomes; Figure S1: Self-regulation funnel plot; Figure S2: Self-regulation comparator subgroup analysis; Figure S3: Self-regulation construct-coverage subgroup analysis; Figure S4: Self-regulation mean-age meta-regression; Figure S5: Self-regulation intervention-duration meta-regression; Figure S6: Functional mean-age meta-regression.
