# Supplementary material for: From Self-Regulation to Function: A Systematic Review and Meta-Analysis of Randomised Controlled Trials in Autistic Children and Young People
Source: Children (Basel). 2026 Oct 15;13(10):1415. doi: 10.3390/children13101415 (PMC13703768; doi:10.3390/children13101415)
Supplement: Supplementary file 1 [file children-13-01415-s001.zip › children-4585768-supplementary.pdf]

Supplementary Material

Supplementary Table S1. Search strategies by database

**Database; Search Engine**	**Search Terms**	**Limitations (confirm word for this)**
MedLine; EBSCO	(((MH "Autism Spectrum Disorder" OR MH "Autistic Disorder" OR MH "Asperger Syndrome" OR MH "Child Development Disorders, Pervasive") AND (AB (self-regulat* OR emotion* regulat* OR affect regulat* OR behavior* regulat* OR behaviour* regulat* OR emotion* dysregulat* OR emotion* disregulat* OR emotion* manag* OR emotion* competenc* OR effortful control OR behavior* manag* OR behaviour* manag*) OR TI (self-regulat* OR emotion* regulat* OR affect regulat* OR behavior* regulat* OR behaviour* regulat* OR emotion* dysregulat* OR emotion* disregulat* OR emotion* manag* OR emotion* competenc* OR effortful control OR behavior* manag* OR behaviour* manag*) OR SU (self-regulat* OR emotion* regulat* OR affect regulat* OR behavior* regulat* OR behaviour* regulat* OR emotion* dysregulat* OR emotion* disregulat* OR emotion* manag* OR emotion* competenc* OR effortful control OR behavior* manag* OR behaviour* manag*))	English; all children 0-18 years
PubMed; PubMed	((("Autism Spectrum Disorder"[MeSH Terms] OR "Autistic Disorder"[MeSH Terms] OR "Asperger Syndrome"[MeSH Terms] OR "Child Development Disorders, Pervasive"[MeSH])) AND (self-regulat*[Title/Abstract] OR emotion* regulat*[Title/Abstract] OR affect regulat*[Title/Abstract] OR behavior* regulat*[Title/Abstract] OR behaviour* regulat*[Title/Abstract] OR emotion* dysregulat*[Title/Abstract] OR emotion* disregulat*[Title/Abstract] OR emotion* manag*[Title/Abstract] OR emotion* competenc*[Title/Abstract] OR effortful control[Title/Abstract] OR behavior* manag*[Title/Abstract] OR behaviour* manag*[Title/Abstract]))	English; child (birth to 18 years)
PsycINFO; Ovid	((Autism Spectrum Disorder or Autism or autis* or asperg* or pervasive) and (self-regulat* or emotion*-regulat* or affect-regulat* or behavior*-regulat* or behaviour-regulat* or emotion*-dysregulat* or emotion*-disregulat* or emotion*-manag* or emotion*-competenc* or effortful-control or behavior*-manag* or behaviour*-manag*)).mp. and ("100" or "120" or "140" or "160" or "180" or "200").ag.	English
ERIC; EBSCO	((SU (Autism Spectrum Disorders or Asperger Syndrome or Aspergers Syndrome) OR TI ("autis*" or "asperg*" or "pervasive development*") OR AB ("autis*" or "asperg*" or "pervasive development*")) AND (TI (self-regulat* or emotion* regulat* or affect regulat* or behavior* regulat* or behaviour* regulat* or emotion* dysregulat* or emotion* disregulat* or emotion* manag* or emotion* competenc* or effortful control or behavior* manag* or behaviour* manag*) OR AB (self-regulat* or emotion* regulat* or affect regulat* or behavior* regulat* or behaviour* regulat* or emotion* dysregulat* or emotion* disregulat* or emotion* manag* or emotion* competenc* or effortful control or behavior* manag* or behaviour* manag*)))	English

Database; Search Engine	Search Terms	Limitations (confirm word for this)
CINAHL; EBSCO	(((MH "Autism Spectrum Disorder” OR MH "Autistic Disorder" OR MH “Asperger Syndrome” OR MH "Pervasive Developmental Disorder-Not Otherwise Specified" OR MH "Child Development Disorders, Pervasive")) AND (AB (self-regulat* OR emotion* regulat* OR affect regulat* OR behavior* regulat* OR behaviour* regulat* OR emotion* dysregulat* OR emotion* disregulat* OR emotion* manag* OR emotion* competenc* OR effortful control OR behavior* manag* OR behaviour* manag*) OR TI (self-regulat* OR emotion* regulat* OR affect regulat* OR behavior* regulat* OR behaviour* regulat* OR emotion* dysregulat* OR emotion* disregulat* OR emotion* manag* OR emotion* competenc* OR effortful control OR behavior* manag* OR behaviour* manag*) OR SU (self-regulat* OR emotion* regulat* OR affect regulat* OR behavior* regulat* OR behaviour* regulat* OR emotion* dysregulat* OR emotion* disregulat* OR emotion* manag* OR emotion* competenc* OR effortful control OR behavior* manag* OR behaviour* manag*))	English; all children

Supplementary Table S2. Principal pooled analyses: full statistical reporting

Outcome	*k*	Hedges' g	*SE*	Variance	95% CI	*Z*	*p*	τ	τ^2	95% prediction interval
Self-regulation	27	0.605	0.077	0.005962	0.454 to 0.756	7.836	< .001	0.270	0.073	0.027 to 1.183
Functional outcomes	25	0.527	0.087	0.007586	0.356 to 0.698	6.049	< .001	0.320	0.102	-0.158 to 1.212
Self-regulation — Hartung–Knapp sensitivity	27	0.605	0.083	—	0.435 to 0.775	$t(26) = 7.31$	< .001	0.270	0.073	—
Functional outcomes — Hartung–Knapp sensitivity	25	0.527	0.096	—	0.328 to 0.725	$t(24) = 5.48$	< .001	0.320	0.102	—

Note. Principal analyses use random-effects models. The functional primary synthesis retains 25 eligible intervention-comparator comparisons from 23 independent studies; the later functional moderator analyses use one effect per independent study. Prediction intervals estimate the range of true effects expected in comparable future studies.

Supplementary Table S3. Sensitivity analyses

Outcome	Sensitivity analysis	*k*	Hedges' *g*	95% CI	τ	Interpretation/test
Self-regulation	Primary analysis	27	0.605	0.454 to 0.756	0.270	PI 0.027 to 1.183
Self-regulation	Exclude high risk of bias	20	0.538	0.400 to 0.677	0.153	Positive and significant
Self-regulation	Exclude active comparator/component flagged comparisons	15	0.818	0.581 to 1.055	0.323	Positive and significant
Self-regulation	Exclude component-only outcomes	21	0.624	0.434 to 0.813	0.318	Positive and significant
Self-regulation	Exclude hybrid SR/function outcomes	17	0.544	0.368 to 0.720	0.208	Positive and significant
Self-regulation	Exclude non-standard/model-derived inputs	22	0.635	0.444 to 0.826	0.324	Positive and significant
Function	Primary analysis	25	0.527	0.356 to 0.698	0.320	PI -0.158 to 1.212
Function	Exclude high risk of bias	20	0.488	0.306 to 0.670	0.297	Positive and significant
Function proximity: active comparators only	Direct/target-aligned	4	0.659	0.220 to 1.099	0.367	$Q_\beta = 3.770$, $p = .052$
Function proximity: active comparators only	Distal/generalisation	7	0.196	0.038 to 0.355	0.000	Positive distal effect

Note. PI = prediction interval; the 95% PI represents the expected range of true effects across comparable future studies. Each sensitivity analysis was rerun independently from the relevant primary dataset. The active-comparator proximity analysis is a targeted stress test of the strongest new functional moderator, not a replacement for the primary functional synthesis.

Supplementary Table S4. Categorical moderator analyses—full statistical reporting

Outcome	Moderator	Group	*k*	*g*	*SE*	95% CI	Group *p*	τ	τ^2	95% PI	Q_β / *p*
SR	Comparator	Active/attention	13	0.444	0.075	0.297 to 0.590	< .001	0.108	0.012	0.154 to 0.733	Q_β = 12.234; p = .002
SR	Comparator	Treatment as usual	5	0.747	0.371	0.021 to 1.474	.044	0.685	0.470	-1.732 to 3.227	
SR	Comparator	Waitlist/no treatment	9	0.878	0.100	0.682 to 1.074	< .001	0.000	0.000	Not estimated	
SR	Construct coverage	Component-level	6	0.553	0.110	0.337 to 0.768	< .001	0.036	0.001	0.231 to 0.874	Q_β = 0.883; p = .643
SR	Construct coverage	Domain-level	9	0.719	0.143	0.438 to 1.000	< .001	0.282	0.080	-0.029 to 1.467	
SR	Construct coverage	Global/multi-domain	12	0.583	0.136	0.317 to 0.849	< .001	0.357	0.127	-0.267 to 1.433	
Function	Outcome proximity	Direct/target-aligned	13	0.761	0.136	0.496 to 1.027	< .001	0.382	0.146	-0.131 to 1.654	Q_β = 11.034; p < .001
Function	Outcome proximity	Distal/ generalisation	10	0.250	0.073	0.108 to 0.393	.001	0.000	0.000	Not estimated	
Function	Supported application + generalisation	No	10	0.409	0.139	0.138 to 0.681	.003	0.324	0.105	-0.403 to 1.222	Q_β = 0.918; p = .338
Function	Supported application + generalisation	Yes	13	0.578	0.109	0.365 to 0.792	< .001	0.290	0.084	-0.104 to 1.260	
Function	Target-skill application/rehearsal	No	8	0.354	0.165	0.031 to 0.678	.032	0.345	0.119	-0.581 to 1.289	Q_β = 1.353; p = .245
Function	Target-skill application/rehearsal	Yes	15	0.578	0.098	0.385 to 0.770	< .001	0.278	0.077	-0.059 to 1.214	
Function	Generalisation support	No	9	0.430	0.154	0.128 to 0.731	.005	0.352	0.124	-0.479 to 1.338	Q_β = 0.622; p = .430
Function	Generalisation support	Yes	13	0.578	0.109	0.365 to 0.792	< .001	0.290	0.084	-0.104 to 1.260	
Function	Comparator	Active/attention	11	0.335	0.097	0.145 to 0.525	.001	0.216	0.047	-0.200 to 0.870	Q_β = 7.167; p = .028
Function	Comparator	Treatment as usual	4	0.727	0.382	-0.021 to 1.476	.057	0.660	0.436	-2.555 to 4.010	
Function	Comparator	Waitlist/no treatment	8	0.739	0.121	0.502 to 0.977	< .001	0.179	0.032	0.210 to 1.269	

Outcome	Moderator	Group	*k*	*g*	*SE*	95% CI	Group *p*	τ	$τ^2$	95% PI	$Q_β$ / *p*
Function	Intervention type	Cognitive/executi ve-function	3	0.471	0.283	-0.084 to 1.026	.096	0.392	0.154	-5.673 to 6.615	**$Q_β$** = 0.487; p = .784
Function	Intervention type	Physical/activity-based	3	0.933	0.628	-0.299 to 2.165	.138	1.007	1.013	-14.145 to 16.011	
Function	Intervention type	Psychosocial/soci al-emotional	17	0.496	0.086	0.327 to 0.665	< .001	0.242	0.059	-0.052 to 1.043	
Function (active comparators)	Outcome proximity	Direct/target-aligned	4	0.659	0.224	0.220 to 1.099	.003	0.367	0.134	-1.190 to 2.508	**$Q_β$** = 3.770; p = .052
Function (active comparators)	Outcome proximity	Distal/generalisat ion	7	0.196	0.081	0.038 to 0.355	.015	0.000	0.000	Not estimated	

Note. $Q_β$ = mixed-effects between-subgroup Q test. Group p values test whether the subgroup pooled effect differs from zero; $Q_β$ tests whether subgroup pooled effects differ from one another. The functional mechanism analyses use 23 independent study-level effects. The generalisation-support analysis uses k = 22 because one unclear classification was treated as missing. Prediction intervals (PI) are shown where CMA returned an estimable interval; extremely wide intervals in sparse intervention-type subgroups reflect low k and substantial between-study variance.

Supplementary Table S5. Meta-regression results

Outcome	Covariate	*k*	β	Approx. SE	95% CI	p	Residual τ	Interpretation
Self-regulation	Mean age	27	-0.027	0.031	-0.088 to 0.035	.391	0.284	No linear age association
Self-regulation	Duration (weeks)	25	0.002	0.009	-0.015 to 0.020	.788	0.300	No linear duration association
Function	Mean age	25	-0.026	0.035	-0.094 to 0.042	.460	0.333	No linear age association
Function	Duration (weeks)	23	0.007	0.024	-0.040 to 0.054	.770	0.360	No linear duration association
Function	Total contact hours	21	0.001	0.002	-0.003 to 0.005	.626	—	Exploratory; no linear contact-hours association

Note. Approximate SE values were derived from the reported 95% confidence intervals where an exact SE was not retained in the exported CMA output and are included to preserve regression reporting detail. Mean age was preferred over categorical age groups because inconsistent labels in an earlier exploratory output created artificial subgroups. Total contact hours were incomplete and remain exploratory.

Supplementary Table S6. Small-study-effect diagnostics for self-regulation and functional outcomes

Diagnostic	Result	Interpretation
**Self-regulation outcomes**		
Begg–Mazumdar rank correlation	Kendall's τ-*b* = 0.182; two-tailed *p* = .182	No statistically significant rank-correlation signal.
Egger regression	Intercept = 1.631; 95% CI -0.080 to 3.343; two-tailed *p* = .061	Suggestive asymmetry signal, but it did not reach the conventional threshold for statistical significance.
Classic fail-safe N	834	Descriptive only; not used as the primary small-study-effect conclusion.
Duval–Tweedie trim-and-fill	Four studies imputed under the trim-and-fill asymmetry model; adjusted *g* = 0.497, 95% CI 0.325 to 0.669	Adjusted effect remained positive under the asymmetry model.
**Functional outcomes** (23 independent study-level effects); multi-arm intervention groups were combined so that each study contributed one effect.		
Begg–Mazumdar rank correlation	Kendall's τ-*b* = 0.399; two-tailed p = .007	Statistically significant funnel-asymmetry signal; not interpreted as proof of publication bias.
Egger regression	Intercept = 3.403; 95% CI 1.356 to 5.451; two-tailed *p* = .002	Statistically significant asymmetry signal; cause of asymmetry cannot be identified by this test.
Classic fail-safe N	519	Descriptive only; not used as the primary reporting-bias conclusion.
Duval–Tweedie trim-and-fill	Six studies imputed on the left; adjusted *g* = 0.357, 95% CI 0.165 to 0.549	Adjusted effect remained positive under the trim-and-fill model; procedure is assumption-dependent.

Supplementary Table S7. Functional mechanism coding definitions

Variable	Operational definition	Inferential status
Functional outcome proximity	Direct/target-aligned when the selected functional domain was explicitly targeted or repeatedly practised; distal/generalisation when the selected functional outcome assessed function beyond the directly trained or practised intervention target.	Primary new functional moderator
Target-skill application/rehearsal	Repeated enactment or deployment of the targeted regulatory, social, cognitive-functional, or behavioural strategy/skill. Repetition of an unrelated activity alone did not qualify.	Secondary moderator
Generalisation support	Explicit strategy intended to transfer learning beyond the immediate training task/session, such as real-life homework, in-vivo assignments, parent/teacher coaching, or home-school transfer procedures.	Secondary moderator; unclear treated as missing
Supported application + generalisation	Derived Yes only when both target-skill application/rehearsal and explicit generalisation support were coded Yes. This was not a summed score.	Principal mechanism moderator
Orientation (4-level)	Acquisition/capacity-focused; combined acquisition + application; contextual application/performance-focused; experiential/activity-based.	Descriptive only; sparse 4-level cells
Explicit functional goal/content	An identifiable everyday activity, social-performance, adaptive, school, participation, or motor-performance goal explicitly formed part of intervention content.	Audit/descriptive only because of strong overlap with proximity
Naturalistic/ecological application	Target skill applied within a meaningful everyday/functional activity or context relevant to real-world use.	Descriptive only because of strong overlap with application
Multi-context implementation	Intervention deliberately practised or reinforced in at least two meaningful contexts.	Supplementary descriptive only; one unclear code
Key-adult implementation/support	Parent, teacher, caregiver, or other everyday-context adult actively prompted, coached, reinforced, or supported skill application.	Descriptive; overlaps strongly with generalisation support
Direct self-regulation targeting	Whether self-regulation, executive function, emotion regulation, or behavioural regulation was an explicit intervention target.	Audit/descriptive only

Supplementary Table S8. Study-level inputs for multi-arm functional moderator analyses

Study	Group	*N*	Mean	*SD*	Use in 23-study moderator dataset
de Vries et al. (2015)	Combined adaptive WM + flexibility training	55	29.209	12.007	Intervention
de Vries et al. (2015)	Mock training control	29	27.400	11.100	Comparator
Marzouki et al. (2022)	Combined technical + game-based aquatic interventions	16	9.750	2.567	Intervention
Marzouki et al. (2022)	Treatment as usual	6	4.000	1.550	Comparator

Note. The principal functional meta-analysis retained separate eligible intervention comparisons with split shared-comparator sample sizes. For the independent-study moderator analyses, arms with identical moderator coding were combined and the original shared comparator was used once, preventing the multi-arm studies from receiving disproportionate weight.

Supplementary Table S9. Final study-level functional moderator coding

Paper	Study	Outcome proximity	Supported application + generalisation	Target-skill application/rehearsal	Generalisation support	Comparator	Intervention type
1	Afsharnejad et al. (2022)	Direct/target-aligned	Yes	Yes	Yes	Active/attention comparator	Psychosocial/social-emotional
2	Beaumont & Sofronoff (2008)	Direct/target-aligned	Yes	Yes	Yes	Waitlist/no treatment	Psychosocial/social-emotional
3	Beaumont et al. (2021)	Direct/target-aligned	Yes	Yes	Yes	Active/attention comparator	Psychosocial/social-emotional
4	Cahart et al. (2022)	Distal/generalisation	No	No	No	Treatment as usual	Cognitive/executive-function training
5	Clifford et al. (2022)	Distal/generalisation	Yes	Yes	Yes	Active/attention comparator	Psychosocial/social-emotional
6	de Vries et al. (2015)	Distal/generalisation	No	No	No	Active/attention comparator	Cognitive/executive-function training
8	Fatta et al. (2024)	Direct/target-aligned	Yes	Yes	Yes	Waitlist/no treatment	Psychosocial/social-emotional
9	Gabriels et al. (2015)	Distal/generalisation	No	No	No	Active/attention comparator	Physical/activity-based
13	Kirst et al. (2022)	Distal/generalisation	Yes	Yes	Yes	Active/attention comparator	Psychosocial/social-emotional
14	Lopata et al. (2010)	Direct/target-aligned	Yes	Yes	Yes	Waitlist/no treatment	Psychosocial/social-emotional
15	Lopata et al. (2015)	Distal/generalisation	No	No	No	Active/attention comparator	Psychosocial/social-emotional
16	Lopata et al. (2019)	Direct/target-aligned	Yes	Yes	Yes	Treatment as usual	Psychosocial/social-emotional
17	Lopata et al. (2021)	Direct/target-aligned	Yes	Yes	Yes	Waitlist/no treatment	Psychosocial/social-emotional
18	Marzouki et al. (2022)	Direct/target-aligned	No	No	No	Treatment as usual	Physical/activity-based
19	Morgan et al. (2018)	Distal/generalisation	No	Yes	No	Active/attention comparator	Psychosocial/social-emotional
22	Soorya et al. (2015)	Direct/target-aligned	Yes	Yes	Yes	Active/attention comparator	Psychosocial/social-emotional
23	Thomeer et al. (2012)	Direct/target-aligned	Yes	Yes	Yes	Waitlist/no treatment	Psychosocial/social-emotional

Paper	Study	Outcome proximity	Supported application + generalisation	Target-skill application/rehearsal	Generalisation support	Comparator	Intervention type
24	Thomeer et al. (2015)	Distal/generalisation	No	No	Missing	Waitlist/no treatment	Psychosocial/social-emotional
25	Thomeer et al. (2019)	Direct/target-aligned	Yes	Yes	Yes	Waitlist/no treatment	Psychosocial/social-emotional
26	Wan Yunus et al. (2021)	Direct/target-aligned	No	Yes	No	Active/attention comparator	Cognitive/executive-function training
27	Wang et al. (2025)	Direct/target-aligned	No	No	No	Treatment as usual	Physical/activity-based
28	Weiss et al. (2018)	Distal/generalisation	Yes	Yes	Yes	Waitlist/no treatment	Psychosocial/social-emotional
29	Williams et al. (2012)	Distal/generalisation	No	No	No	Active/attention comparator	Psychosocial/social-emotional

Note. "Missing" denotes insufficient intervention reporting and was not entered as a third subgroup. Coding definitions and the analysis hierarchy were finalised before the corresponding moderator results were inspected.

Supplementary Table S10. Final inferential status of functional mechanism variables

Variable	Final status	Reason
Functional outcome proximity	Primary exploratory moderator	Balanced 13/10 split; directly tests target-aligned versus distal function.
Supported application + generalisation	Principal mechanism moderator	Theory-driven conjunction; tested as Yes/No.
Target-skill application/rehearsal	Secondary moderator	Tested as Yes/No; overlaps with generalisation support.
Generalisation support	Secondary moderator	Tested as Yes/No with one Unclear study omitted.
Comparator type	Design moderator and confounding check	Significant design feature; used to stress-test proximity.
Intervention type	Exploratory contextual moderator	Broad categories; no evidence of moderation.
Orientation (4-level)	Descriptive only	Sparse cell: contextual application/performance k = 1.
Explicit functional goal/content	Descriptive only	Near-deterministic overlap with outcome proximity.
Naturalistic/ecological application	Descriptive only	Strong overlap with target-skill application.
Multi-context implementation	Supplementary descriptive only	Conceptual overlap and one Unclear code; no formal conclusion retained.
Key-adult implementation/support	Descriptive only	Strong overlap with generalisation support and incomplete reporting.
Generalisation adherence assessed	Audit only	Too sparsely reported for analysis.
Mechanism score (0–4)	Not analysed	Equal weighting and unidimensionality were not defensible.

Supplementary Table S11. Self-regulation construct alignment

Element	Final review specification	Alignment with Janicaud et al. (2026)
Umbrella construct	Self-regulation treated as regulation of cognitive processes/thoughts, behaviour, and emotion in service of goal-directed action.	Aligned with cognitive, behavioural, and emotional regulation as broad domains.
Outcome eligibility	Trials could contribute an outcome representing one SR domain or component; measures were not required to span all three domains.	Preserves intervention evidence while acknowledging that many measures incompletely sample the multidimensional construct.
Construct-coverage coding	Component-level, domain-level, and global/multi-domain.	Makes degree of construct coverage explicit rather than treating all SR measures as equivalent.
Construct-coverage moderator	No evidence of differential pooled effect by construct coverage (Q_β = 0.883, p = .643).	Supports robustness of the intervention signal across construct-coverage levels, not psychometric equivalence of measures.
Future measurement	Pair proximal SR outcomes with target-aligned and distal functional outcomes; specify SR component/domain coverage.	Consistent with calls for more comprehensive and ecologically contextualised SR assessment.

Supplementary Table S12. Protocol-to-final-review summary

Review element	Registered protocol	Final review	Rationale/status
Eligible study design	Randomised and quasi-experimental designs were eligible.	Restricted to randomised and cluster-randomised controlled trials.	Restriction increased design comparability and aligned the quantitative synthesis with randomised evidence.
Intervention scope	Pharmacological/medical exclusions were not explicitly specified.	Review explicitly excluded pharmacological and medical interventions.	Maintained the review focus on non-pharmacological self-regulation-focused interventions.
Functional outcome eligibility	Functional outcomes were not specified as a main outcome in the registered record.	At least one separate child functional outcome was required for inclusion.	The final review was designed to distinguish self-regulation change from child functioning.
Functional proximity and mechanism analyses	Not prespecified.	Added as theory-driven exploratory analyses; coding definitions and analysis hierarchy were finalised before corresponding moderator results were inspected.	Exploratory extension developed to examine transfer/generalisation and potential intervention mechanisms.

Supplementary Table S13. Outcome-selection decisions for studies with multiple eligible outcomes

Study	Outcome stream requiring hierarchy	Selected outcome	Selection note
Afsharnejad et al. (2022)	Self-regulation and function	SR: ERSSQ-C total; Function: Goal Attainment Scaling goals	ERSSQ-C selected for alignment with intervention target; GAS selected as the clearest everyday functional/social-goal outcome, distinct from the SR outcome.
Beaumont & Sofronoff (2008)	Self-regulation	ERSSQ-P total	Broader outcome measure selected over narrower direct task outcomes. Parent report selected consistently where the same construct was available from multiple informants.
Clifford et al. (2022)	Self-regulation	BARQ-P Adaptive Strategies	Selected as the measure most directly represented the intervention's anger-regulation target. Parent report selected consistently where the same construct was available from multiple informants.
de Vries et al. (2015)	Self-regulation	BRIEF Global Executive Composite (both intervention arms)	Broader validated measure selected over narrower executive-function task outcomes.
Kenworthy et al. (2014)	Self-regulation	BRIEF Shift	Selected as the broader validated outcome measure most directly aligned with the intervention's target.
Kirst et al. (2022)	Self-regulation	ERC Emotion Regulation subscale	Selected for direct alignment with emotional regulation rather than narrower recognition/awareness tasks.
Lopata et al. (2010)	Self-regulation	Adapted Skillstreaming Checklist total	Broader validated intervention-relevant outcome selected.
Lopata et al. (2015)	Self-regulation	Adapted Skillstreaming Checklist total	Broader validated intervention-relevant outcome selected.
Lopata et al. (2019)	Self-regulation	Adapted Skillstreaming Checklist total	Broader validated intervention-relevant outcome selected.
Lopata et al. (2021)	Self-regulation and function	Adapted Skillstreaming Checklist total; Function: BASC-3-PRS social skills scale	SR broader validated intervention-relevant outcome selected; BASC-3-PRS selected as the broad social skills function measure.
Morgan et al. (2018)	Function	VABS-2 Adaptive Behaviour Composite	Selected as the broad validated everyday adaptive-function measure.
Soorya et al. (2015)	Self-regulation	DANVA-2 total	Selected as the distinct social-cognitive/self-regulation outcome; CCC-2 Social Relations was retained as the separate functional outcome.
Thomeer et al. (2012)	Self-regulation	Adapted Skillstreaming Checklist total	Broader validated intervention-relevant outcome selected.
Thomeer et al. (2015)	Self-regulation	CAM-C Faces total	Selected as the primary direct emotion-recognition outcome aligned with the intervention target.
Wan Yunus et al. (2021)	Function	School Function Assessment, Part III: Activity Performance — Cognitive/Behaviour Tasks	Selected as the school-function outcome representing everyday task performance.
Weiss et al. (2018)	Self-regulation	ERSSQ-P total	Selected as the parent-reported measure most directly aligned with the intervention's emotion-regulation target.
Williams et al. (2012)	Self-regulation	NEPSY-II Affect Recognition	Selected as the principal emotion-recognition outcome for the intervention target.

Supplementary Figures

Supplementary Figure S1. Self-regulation funnel plot.

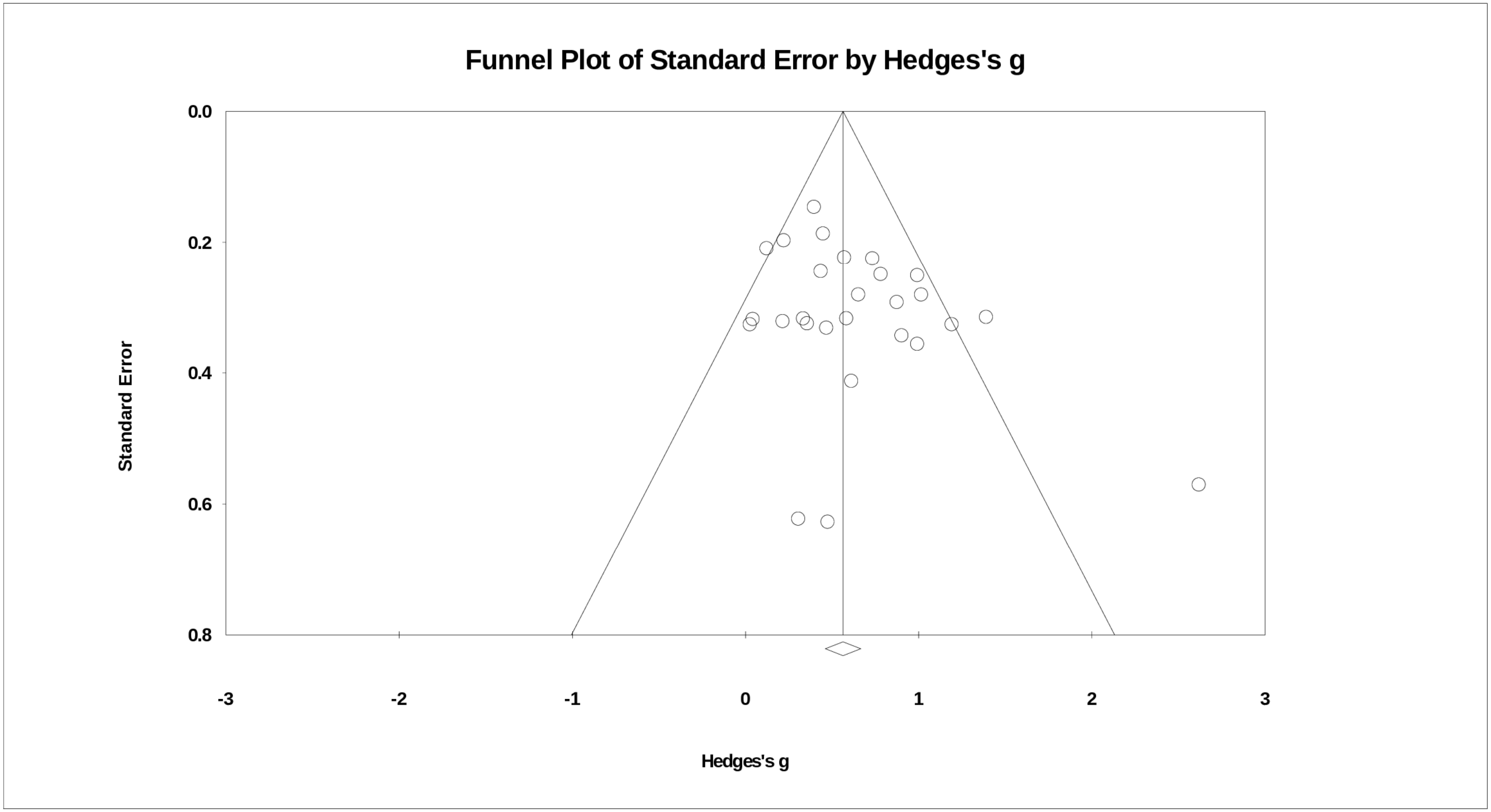

Note. Funnel plot for the self-regulation synthesis. Interpret alongside the Begg–Mazumdar rank-correlation test, Egger regression, fail-safe N, and trim-and-fill diagnostics reported in Supplementary Table S6.

Supplementary Figure S2. Self-regulation comparator subgroup analysis.

Comparator Moderator Analysis

Study name	Group by Comparator	Hedges's g	Standard error	Variance	Lower limit	Upper limit	Z-Value	p-Value
		Statistics for each study						
1. Afsharnejad et al. (2022)	Active/attention comparator	0.122	0.209	0.044	-0.288	0.532	0.584	0.559
3. Beaumont et al. (2021)	Active/attention comparator	0.992	0.251	0.063	0.500	1.483	3.955	0.000
5. Clifford et al. (2022)	Active/attention comparator	0.873	0.292	0.085	0.301	1.446	2.991	0.003
6a. de Vries et al. (2015)	Active/attention comparator	0.042	0.318	0.101	-0.581	0.665	0.132	0.895
6b. de Vries et al. (2015)	Active/attention comparator	0.215	0.321	0.103	-0.414	0.844	0.669	0.504
9. Gabriels et al. (2015)	Active/attention comparator	0.448	0.187	0.035	0.082	0.814	2.398	0.016
11. Kenworthy et al. (2014)	Active/attention comparator	0.651	0.280	0.079	0.102	1.201	2.324	0.020
13. Kirst et al. (2022)	Active/attention comparator	0.570	0.223	0.050	0.133	1.008	2.554	0.011
15. Lopata et al. (2015)	Active/attention comparator	0.025	0.326	0.106	-0.614	0.664	0.077	0.938
19. Morgan et al. (2018)	Active/attention comparator	0.396	0.146	0.021	0.109	0.682	2.707	0.007
22. Soorya et al. (2015)	Active/attention comparator	0.435	0.245	0.060	-0.045	0.914	1.776	0.076
26. Wan Yunus et al. (2021)	Active/attention comparator	0.583	0.317	0.100	-0.038	1.203	1.840	0.066
29. Williams et al. (2012)	Active/attention comparator	0.333	0.317	0.100	-0.288	0.954	1.050	0.294
Pooled	Active/attention comparator	0.444	0.075	0.006	0.297	0.590	5.930	0.000
4. Cahart et al. (2022)	Treatment as usual	0.467	0.331	0.110	-0.182	1.116	1.410	0.158
16. Lopata et al. (2019)	Treatment as usual	0.221	0.197	0.039	-0.166	0.607	1.120	0.263
18a. Marzouki et al. (2022)	Treatment as usual	0.305	0.622	0.387	-0.915	1.525	0.490	0.624
18b. Marzouki et al. (2022)	Treatment as usual	0.475	0.627	0.393	-0.755	1.704	0.757	0.449
27. Wang et al. (2025)	Treatment as usual	2.618	0.570	0.325	1.500	3.736	4.589	0.000
Pooled	Treatment as usual	0.747	0.371	0.137	0.021	1.474	2.016	0.044
2. Beaumont & Sofronoff (2008)	Waitlist/no treatment	1.390	0.315	0.099	0.773	2.006	4.416	0.000
7. Factor et al. (2019)	Waitlist/no treatment	0.611	0.412	0.170	-0.197	1.419	1.482	0.138
8. Fatta et al. (2024)	Waitlist/no treatment	0.356	0.324	0.105	-0.280	0.992	1.096	0.273
14. Lopata et al. (2010)	Waitlist/no treatment	0.902	0.343	0.118	0.230	1.573	2.630	0.009
17. Lopata et al. (2021)	Waitlist/no treatment	0.733	0.225	0.051	0.292	1.174	3.261	0.001
23. Thomeer et al. (2012)	Waitlist/no treatment	0.992	0.356	0.127	0.294	1.689	2.787	0.005
24. Thomeer et al. (2015)	Waitlist/no treatment	1.191	0.326	0.106	0.552	1.829	3.655	0.000
25. Thomeer et al. (2019)	Waitlist/no treatment	1.015	0.280	0.079	0.465	1.564	3.618	0.000
28. Weiss et al. (2018)	Waitlist/no treatment	0.781	0.249	0.062	0.293	1.269	3.136	0.002
Pooled	Waitlist/no treatment	0.878	0.100	0.010	0.682	1.074	8.775	0.000
Pooled	Overall	0.603	0.059	0.003	0.487	0.719	10.198	0.000

Hedges's g and 95% CI

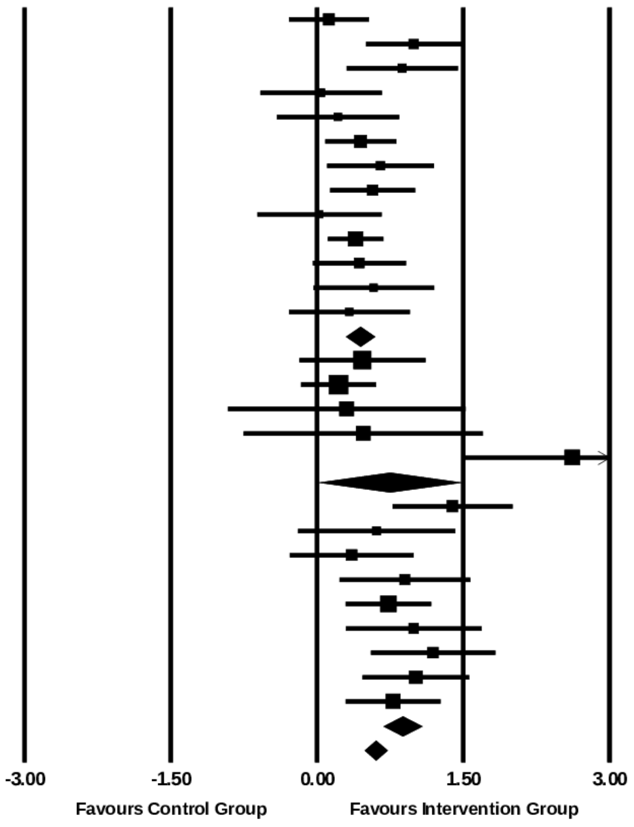

Note. Mixed-effects subgroup analysis by comparator type. Numerical subgroup estimates and the Q_{β} test are reported in Supplementary Table S4.

Supplementary Figure S3. Self-regulation construct-coverage subgroup analysis.

Self-Regulation Construct Coverage Moderator Analysis

Study name	Group by Construct coverage	Std diff in means	Standard error	Variance	Lower limit	Upper limit	Z-Value	p-Value
**Component-level SR**								
4. Cahart et al. (2022)	Component-level SR	0.477	0.339	0.115	-0.186	1.141	1.410	0.158
9. Gabriels et al. (2015)	Component-level SR	0.451	0.188	0.035	0.082	0.820	2.398	0.016
11. Kenworthy et al. (2014)	Component-level SR	0.660	0.284	0.081	0.103	1.217	2.324	0.020
22. Soorya et al. (2015)	Component-level SR	0.440	0.248	0.061	-0.045	0.925	1.776	0.076
24. Thomeer et al. (2015)	Component-level SR	1.213	0.332	0.110	0.563	1.864	3.655	0.000
29. Williams et al. (2012)	Component-level SR	0.340	0.324	0.105	-0.294	0.974	1.050	0.294
Pooled (component-level)	Component-level SR	0.553	0.110	0.012	0.337	0.768	5.020	0.000
Prediction interval	Component-level SR	0.553			0.231	0.874		
**Domain-level SR**								
1. Afsharnejad et al. (2022)	Domain-level SR	0.123	0.211	0.045	-0.290	0.537	0.584	0.559
2. Beaumont & Sofronoff (2008)	Domain-level SR	1.412	0.320	0.102	0.785	2.039	4.416	0.000
3. Beaumont et al. (2021)	Domain-level SR	1.003	0.254	0.064	0.506	1.500	3.955	0.000
5. Clifford et al. (2022)	Domain-level SR	0.887	0.297	0.088	0.306	1.469	2.991	0.003
7. Factor et al. (2019)	Domain-level SR	0.634	0.428	0.183	-0.204	1.472	1.482	0.138
13. Kirst et al. (2022)	Domain-level SR	0.576	0.225	0.051	0.134	1.018	2.554	0.011
18a. Marzouki et al. (2022)	Domain-level SR	0.334	0.681	0.463	-1.001	1.668	0.490	0.624
18b. Marzouki et al. (2022)	Domain-level SR	0.519	0.686	0.471	-0.825	1.864	0.757	0.449
28. Weiss et al. (2018)	Domain-level SR	0.790	0.252	0.063	0.296	1.284	3.136	0.002
Pooled (domain-level)	Domain-level SR	0.719	0.143	0.021	0.438	1.000	5.012	0.000
Prediction interval	Domain-level SR	0.719			-0.029	1.467		
**Global/multi-domain SR**								
6a. de Vries et al. (2015)	Global/multi-domain SR	0.043	0.324	0.105	-0.592	0.677	0.132	0.895
6b. de Vries et al. (2015)	Global/multi-domain SR	0.219	0.328	0.107	-0.423	0.861	0.669	0.504
8. Fatta et al. (2024)	Global/multi-domain SR	0.364	0.332	0.110	-0.286	1.014	1.096	0.273
14. Lopata et al. (2010)	Global/multi-domain SR	0.922	0.351	0.123	0.235	1.609	2.630	0.009
15. Lopata et al. (2015)	Global/multi-domain SR	0.026	0.333	0.111	-0.628	0.679	0.077	0.938
16. Lopata et al. (2019)	Global/multi-domain SR	0.222	0.199	0.039	-0.167	0.612	1.120	0.263
17. Lopata et al. (2021)	Global/multi-domain SR	0.740	0.227	0.051	0.295	1.185	3.261	0.001
19. Morgan et al. (2018)	Global/multi-domain SR	0.397	0.147	0.022	0.110	0.685	2.707	0.007
23. Thomeer et al. (2012)	Global/multi-domain SR	1.016	0.364	0.133	0.301	1.730	2.787	0.005
25. Thomeer et al. (2019)	Global/multi-domain SR	1.029	0.284	0.081	0.471	1.586	3.618	0.000
26. Wan Yunus et al. (2021)	Global/multi-domain SR	0.594	0.323	0.104	-0.039	1.228	1.840	0.066
27. Wang et al. (2025)	Global/multi-domain SR	2.721	0.593	0.352	1.559	3.883	4.590	0.000
Pooled (global/multi-domain)	Global/multi-domain SR	0.583	0.136	0.018	0.317	0.849	4.299	0.000
Prediction interval	Global/multi-domain SR	0.583			-0.267	1.433		
Pooled (overall)	Overall	0.605	0.073	0.005	0.461	0.749	8.241	0.000
Prediction interval	Overall	0.605			0.018	1.192		

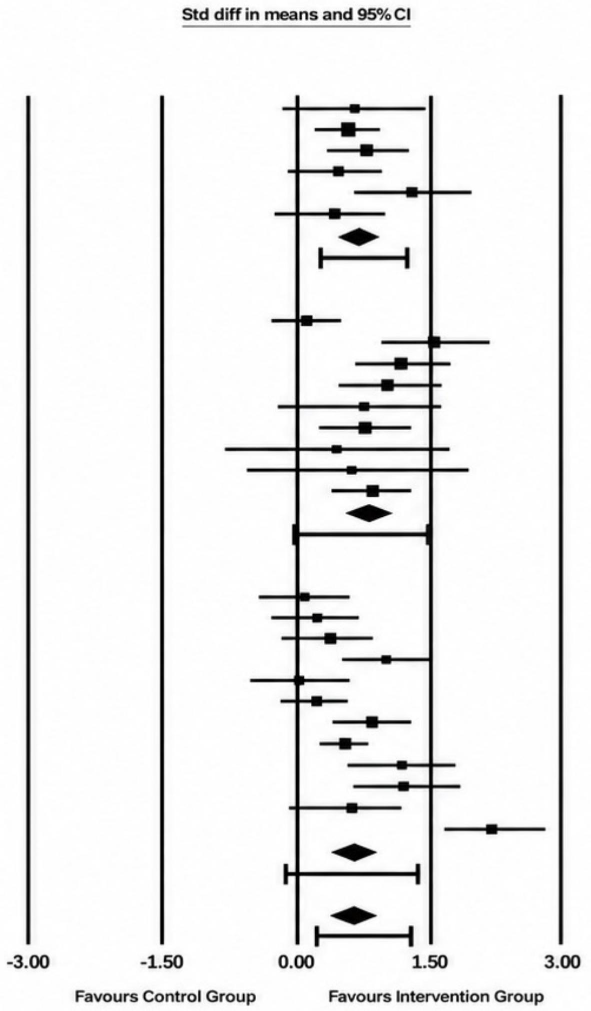

Note. Mixed-effects subgroup analysis of self-regulation construct coverage, comparing component-level, domain-level, and global/multi-domain outcomes. Numerical subgroup estimates and the Q_{β} test are reported in Supplementary Table S4.

Supplementary Figure S4. Self-regulation mean-age meta-regression.

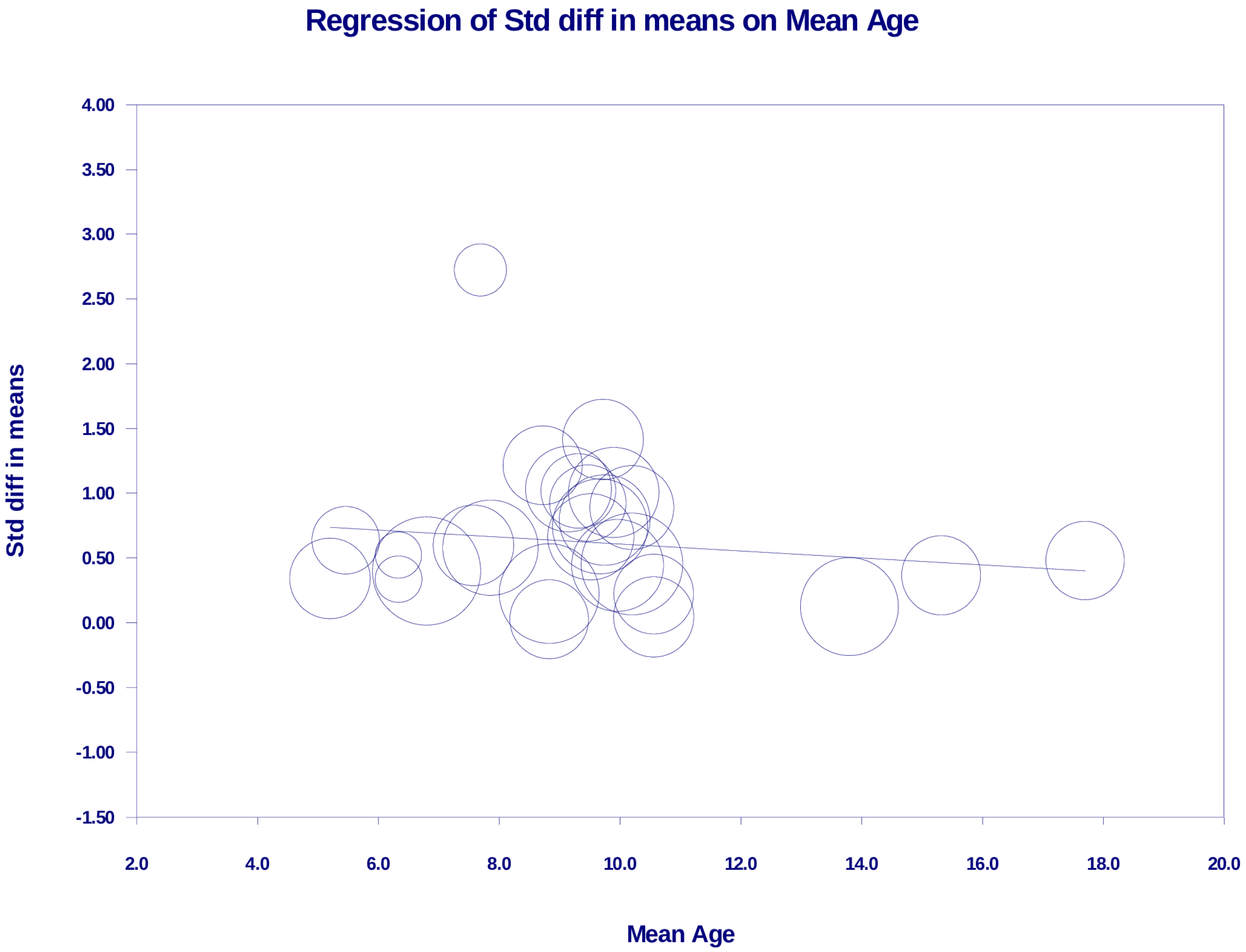

Note. Scatterplot with fitted meta-regression line; numerical model results are reported in Supplementary Table S5.

Supplementary Figure S5. Self-regulation intervention-duration meta-regression.

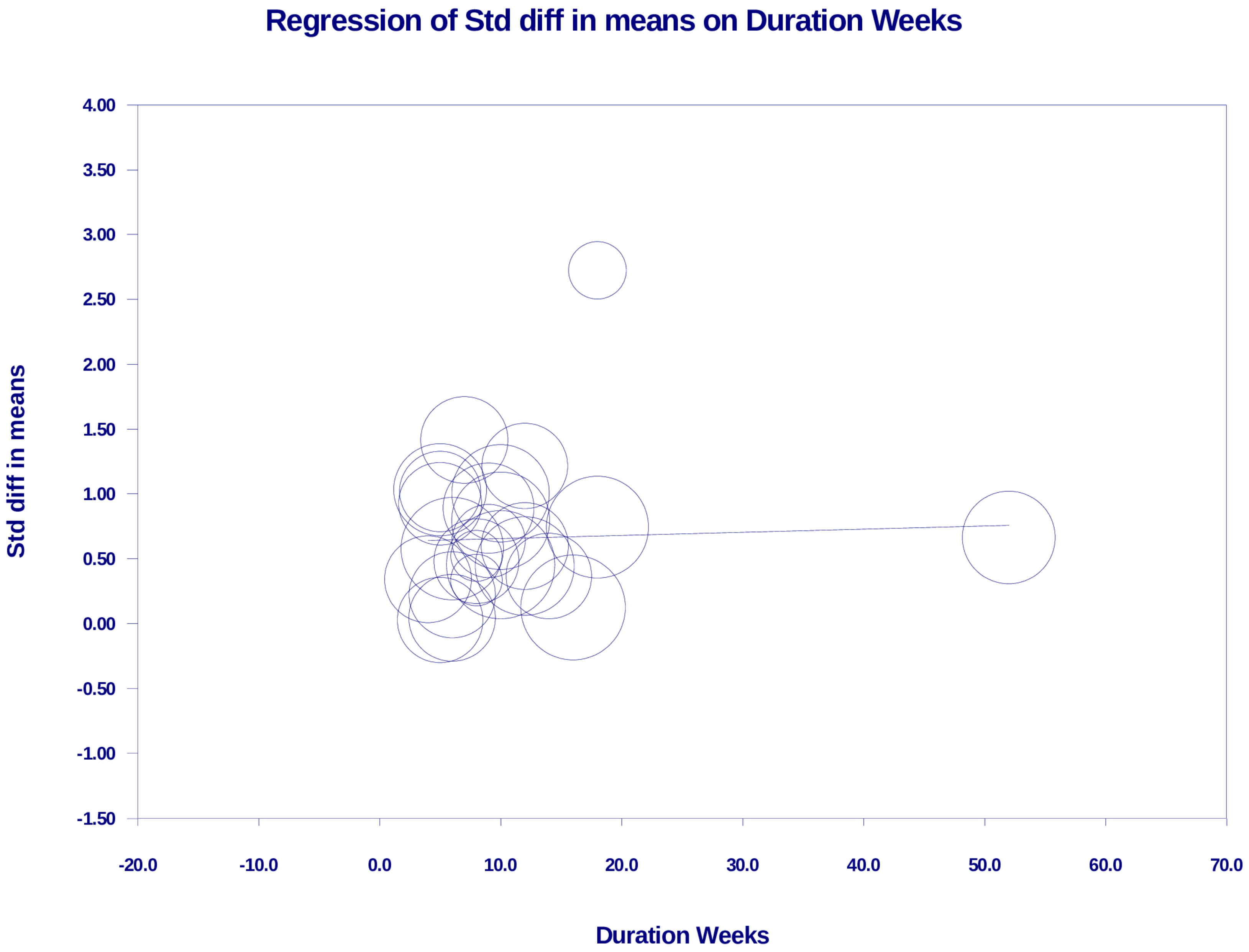

***Note*.** Scatterplot with fitted meta-regression line; duration was available for 25 of 27 comparisons. Numerical model results are reported in Supplementary Table S5.

Supplementary Figure S6. Functional mean-age meta-regression.

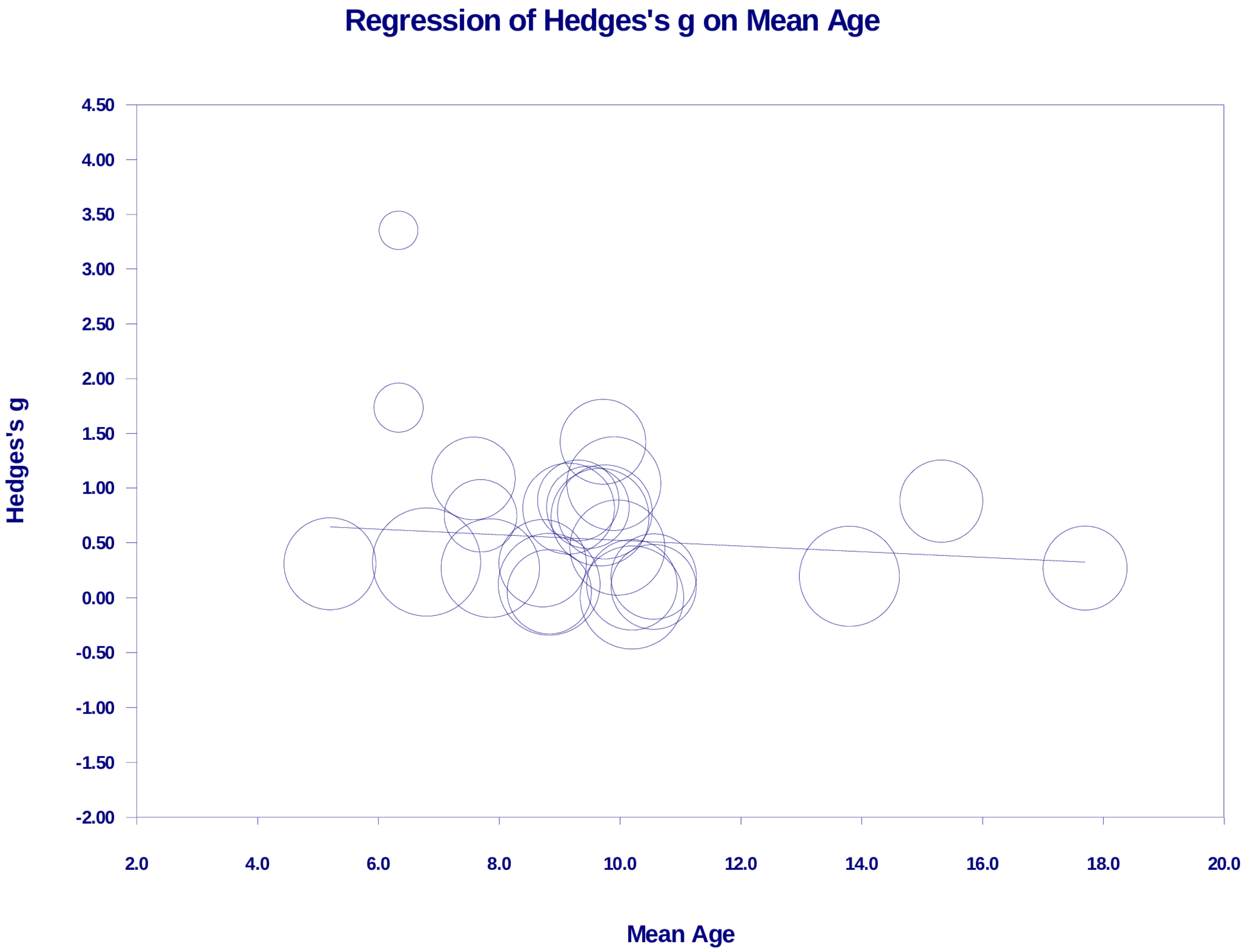

Note. Scatterplot with fitted meta-regression line; numerical model results are reported in Supplementary Table S5.
